# Instant Cascara Beverages with Inulin-Type Carriers: Production Yield, In Vitro Biological Activity and Receptor-Level Responses

**DOI:** 10.3390/nu18121932

**Published:** 2026-06-15

**Authors:** Vanesa Sánchez-Martín, Marta B. López-Parra, Margriet Roelse, Amaia Iriondo-DeHond, Paloma Morales, Ana I. Haza, Maarten A. Jongsma, María Dolores del Castillo

**Affiliations:** 1 Instituto de Investigación en Ciencias de la Alimentación (CIAL), Consejo Superior de Investigaciones Científicas (CSIC), Universidad Autónoma de Madrid (UAM), 28049 Madrid, Spain; vanesa.s@csic.es (V.S.-M.); martab.lopez.parra@gmail.com (M.B.L.-P.); 2 Business Unit Bioscience, Wageningen Plant Research, Droevendaalsesteeg 1, 6708 PB Wageningen, The Netherlands; margriet@insectsense.com (M.R.); maarten.jongsma@wur.nl (M.A.J.); 3 Sección Departamental de Nutrición y Ciencia de los Alimentos, Departamento de Nutrición y Ciencia de los Alimentos, Facultad de Veterinaria, Universidad Complutense, 28040 Madrid, Spain; amaiairi@ucm.es (A.I.-D.); pmorales@ucm.es (P.M.); hanais@ucm.es (A.I.H.)

**Keywords:** biological activities, coffee cherry pulp, Instant Cascara 2.0, inulin-based carriers, receptor-level responses, spray-drying

## Abstract

**Background:** Instant Cascara (IC) beverages, derived from dried coffee cherry pulp, represent an upcycled plant-based ingredient rich in phenolic compounds and methylxanthines. Although spray-drying enables the production of soluble cascara powders without carriers, previous sensory evaluation highlighted limitations in palatability, supporting the need for formulation strategies. **Objective:** To evaluate how the incorporation of inulin-type carriers with different degrees of polymerization modulates production yield, the apparent recovery of bioactive compounds, and formulation-dependent in vitro biological and receptor-level responses of Instant Cascara beverages. **Methods:** Formulations without carrier (IC 0.0) and with long-chain inulin (IC 1.0) or oligofructose-enriched inulin (IC 2.0) were prepared and characterized. Production yield, phytochemical composition, and in vitro antioxidant, anti-inflammatory, antiproliferative, and receptor-mediated responses were assessed using analytical tools, cell-based assays, and receptor-based platforms. **Results:** Carrier incorporation improved production yield, particularly for IC 1.0. Although differences in apparent recovery of bioactive compounds were observed, all formulations preserved relevant in vitro biological activities. IC 2.0 showed stronger nitric oxide inhibition and apoptosis induction in colorectal cancer cell models. Receptor-based assays revealed formulation-dependent differences, including reduced activation of bitter taste receptors (TAS2Rs), absence of sweet receptor (TAS1R2/TAS1R3) activation, and modulation of muscarinic (M3) and dopaminergic (D3/D4) receptor responses. These effects are consistent with variations in the composition and effective concentration of bioactive compounds between formulations, particularly caffeine. **Conclusions:** The incorporation of inulin-type carriers influences production yield and modulates in vitro biological responses and receptor-level responses of Instant Cascara beverages. IC 2.0 represents a formulation with a favorable balance between technological performance and functional responses, associated with a distinct receptor-level profile. This balance may be related to a reduced contribution of bitterness-associated compounds, such as caffeine, together with the preservation of other bioactive components contributing to the observed biological responses. These findings provide a mechanistic in vitro basis for future sensory and in vivo studies evaluating how formulation-dependent differences in bioactive composition may influence physiological responses and consumer perception.

## 1. Introduction

Coffee cherry pulp (cascara) is an authorized novel food and a source of bioactive compounds, including phenolics and methylxanthines, with reported antioxidant and anti-inflammatory activities [1,2,3]. Its upcycling into instant powders aligns with sustainability goals and has been explored in relation to gastrointestinal health and colorectal cancer-related pathways in previous studies [4,5,6,7]. Recent work has also suggested that instant cascara beverages may influence neuroimmune responses along the brain–gut axis in a sex-dependent manner without adverse effects [7]. Spray-drying has been identified as an efficient technology for producing cascara powders, also referred to as Instant Cascara (IC 0.0) [2], but the incorporation of carrier agents adds a formulation variable that can affect product recovery, composition, and downstream responses [8].

Instant Cascara can be produced without carrier agents, but sensory evaluations in human consumers have indicated limitations in palatability at higher concentrations, supporting the need for reformulation strategies [2]. Food-grade carriers are used primarily as processing aids to improve drying behavior and stabilize sensitive compounds during production and storage, rather than to exert direct physiological effects at the consumed dose. Nevertheless, carrier incorporation may influence the microenvironment of native cascara compounds and thereby modify the apparent recovery of bioactive constituents and the expression of in vitro responses. Inulin-type fructans are particularly relevant in this regard because their physicochemical properties may alter matrix interactions during formulation and analysis.

Among these carriers, long-chain inulin (HPX) and oligofructose-enriched inulin (Synergy1) differ in degree of polymerization and solubility, which may lead to distinct effects on spray-drying behavior and formulation properties [9,10,11]. These differences may translate into changes in production yield and the apparent recovery of selected bioactive compounds, while also providing a basis to explore receptor-level responses under controlled in vitro conditions [12]. Previous sensory evaluations of Instant Cascara highlighted the need for reformulation to improve consumer acceptance [2], supporting the relevance of carrier selection for formulation development. In addition, inulin-type fructans are non-digestible carbohydrates and have been associated with reduced post-prandial glycemic impact, consistent with EFSA health claims, which makes them relevant ingredients in food formulation [13]. In the present study, however, their role is considered primarily from a formulation perspective rather than as a direct source of biological activity.

**Figure 1:**
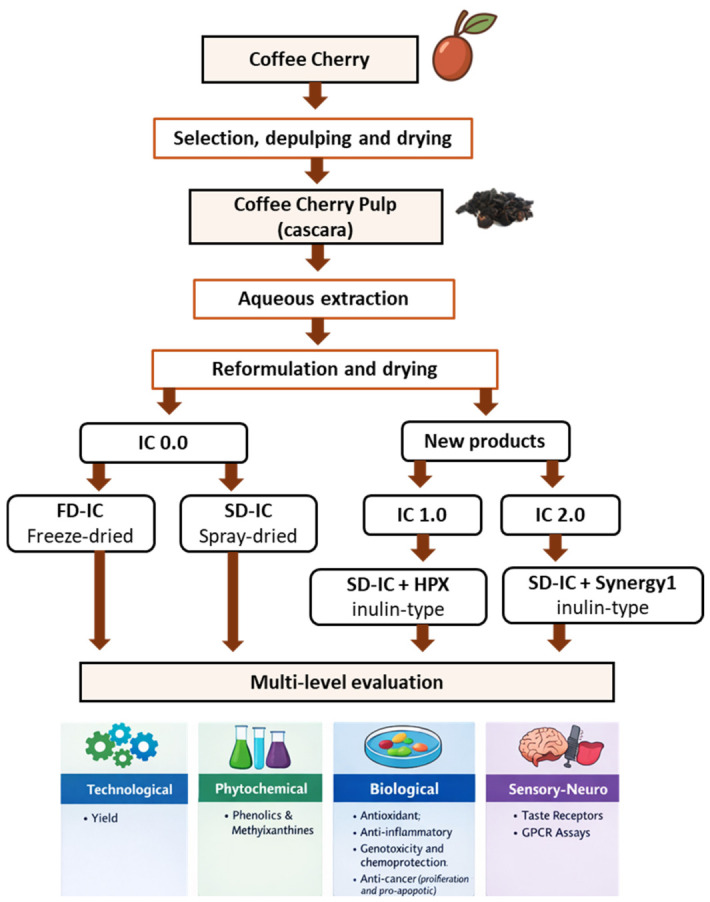
Workflow of Instant Cascara (IC 0.0 (FD-IC), IC 0.0 (SD-IC), IC 1.0 and IC 2.0) production, formulation, and multi-level evaluation. Coffee cherry pulp (cascara) was processed, extracted, and formulated into freeze-dried (IC 0.0 (FD-IC)), spray-dried (IC 0.0 (SD-IC)), and inulin-based carrier-containing powders (HPX and Synergy1, IC 1.0 and IC 2.0, respectively). The resulting products were assessed through an integrated approach including technological performance, phytochemical characterization, and in vitro biological activity and sensory–neurotransmitter receptor responses. This design enables evaluation of the added value of carrier incorporation beyond processing efficiency.

In addition, receptor-based approaches provide complementary mechanistic information by assessing direct interactions between food matrices and specific receptors under controlled conditions. Unlike cell-based biological assays, these systems do not simulate digestion or physiological exposure, but they are useful for exploring ligand–receptor interactions and generating preliminary hypotheses about formulation-dependent effects. To provide an overview of the experimental design and analytical workflow, a schematic representation of the study is shown in Figure 1.

The aim of this study was to evaluate how the incorporation of inulin-type carriers with different degrees of polymerization influences production yield, the apparent recovery of bioactive compounds, and the formulation-dependent in vitro biological and receptor-level responses of Instant Cascara beverages, as a preliminary mechanistic basis for future sensory and in vivo validation.

For clarity, the different Instant Cascara formulations and their corresponding abbreviations are summarized in Table 1. The concentrations used in the different experimental assays were selected to explore mechanistic responses and do not necessarily reflect physiological exposure levels under realistic consumption conditions.

## 2. Materials and Methods

The primary objective of the study was to evaluate the effect of inulin-type carriers on the spray-drying yield and phytochemical profile of Instant Cascara, while secondary analyses assessed formulation-dependent in vitro biological activities and receptor-level responses.

### 2.1. Chemicals and Reagents

All chemicals were of the highest grade commercially available. Benzo(a)pyrene (BaP), dimethyl sulfoxide (DMSO), GelRed®1X, low-melting-point agarose (LMP), Triton X-100, tert-butyl hydroperoxide (tBOOH), vitamin C, 2,7-dichlorofluorescein diacetate (DCFH-DA), 3-(4,5-dimethylthiazol-2-yl)-2,5-diphenyltetrazolium bromide (MTT), lipopolysaccharide (LPS) from *Escherichia coli* O55:B5, nitric oxide (NO), paraformaldehyde, crystal violet, sodium citrate, etoposide, propidium iodide (PI) and RNase A were purchased from Sigma-Aldrich (St. Louis, MO, USA). Ethanol, methanol, EDTA, Tris, sodium hydroxide and sodium chloride were obtained from Panreac Química (Barcelona, Spain). Dulbecco’s modified Eagle Medium (DMEM), L-Glutamine, penicillin and streptomycin, and trypsin were purchased from Gibco Laboratory (Invitrogen Co, Grand Island, NY, USA) and fetal bovine serum (FBS) was from Hyclone (GE Healthcare, Chicago, IL, USA). eBioscience™ Annexin V-FITC Apoptosis Detection Kit and slides (Diagnostic microscope slides, 3-well 14 mm, ER-203B-CE24) were obtained from Invitrogen, Thermo Fisher Scientific (Waltham, MA, USA).

### 2.2. Raw Materials and Formulation

Instant Cascara without carrier (IC 0.0) was produced by both freeze-drying (FD-IC) and spray-drying (SD-IC). Carrier-containing formulations (HPX and Synergy1, referred to as IC 1.0 and IC 2.0, respectively) were obtained exclusively by spray-drying, which is the standard and most efficient process for producing this type of plant-based powder. The different Instant Cascara formulations are defined in Table 1. Previous studies have demonstrated that key functional properties of Instant Cascara, including its antioxidant and anti-inflammatory activities, are preserved during spray-drying, supporting the selection of this process given its favorable economic and environmental profile [2].

IC 0.0 (FD-IC) and IC 0.0 (SD-IC) were used either independently or in comparison, depending on the objective of each assay, to assess the effects of different drying technologies on the native behavior of Instant Cascara. In analyses specifically focused on evaluating the impact of inulin-type carrier incorporation on production yield, in vitro biological activity, and receptor-level responses, IC 0.0 (SD-IC) was used as the reference control, as it shares the same drying process and formulation matrix as the carrier-containing samples. This experimental design enables assessment of whether carrier addition, commonly applied in spray-drying, provides added functional value beyond improvements in processing yield.

#### 2.2.1. Coffee Cherry Pulp (Cascara)

Coffee fruit cascara (skin and pulp of the coffee cherry) from *Coffea arabica* (Tabi variety) was sourced from Colombia and provided by Supracafé S.A. (Móstoles, Madrid, Spain). Cherries were selected using an advanced optical and X-ray sorting system (Multiscan Technologies SLU, Alicante, Spain) to ensure uniform maturity and quality. Cascara was obtained through wet processing, sun-dried, and sanitized by ionizing radiation. Microbiological quality and mycotoxin levels were assessed in accordance with Commission Implementing Regulation (EU) 2022/47 to ensure product safety [1].

#### 2.2.2. Inulin-Based Carriers (HPX and Synergy1)

The present study used long-chain inulin (Orafti® HPX) and oligofructose-enriched inulin (Orafti® Synergy1) as carriers. Both carriers are commercially available, food-grade ingredients were widely used in the preparation of plant-based powders. Their safety, composition, and technological functionality are well established by the manufacturer, and the corresponding product information sheets (PIS) are provided as Supplementary Materials. They were supplied by Beneo GmbH (Mannheim, Germany).

The purpose of carrier incorporation in this study was to evaluate how inulin-type fructans with different degrees of polymerization modulate the technological behavior and functional profile of Instant Cascara. The carriers were primarily considered as processing aids, although their potential contribution to biological responses cannot be excluded.

#### 2.2.3. Preparation of Instant Cascara Powders by Spray-Drying and Freeze-Drying

As previously described, aqueous extracts of dried ripe coffee cherry pulp were obtained by aqueous extraction [2]. Freeze-drying Instant Cascara (IC 0.0 (FD-IC)), used as reference in the present study, was obtained at 0.019 mBar and 21 °C, in a Delta 2-24 LSCplus freeze dryer (CHRIST, Ottobeuren, Germany). Spray-drying Instant Cascara (IC 0.0 (SD-IC)) was performed in a MiniSprayDryer S-300 apparatus (Büchi, Barcelona, Spain) at an air inlet temperature ranging from 150 °C to 170 °C, and outlet air temperatures ranged from 85 °C to 110 °C. Before spray-drying, 3% *w*/*v* inulin-type HPX or Synergy1 was added to the aqueous extract. The microbial quality of the products was evaluated to guarantee their safety before further analysis [2].

The resulting powders represent the final Instant Cascara food ingredients evaluated in this study.

#### 2.2.4. Yield Calculation and Technological Performance

The efficiency of the spray-drying process for instant powders incorporating inulin, Instant Cascara 1.0 and 2.0, was determined by comparing the experimental and theoretical values of the resulting soluble powder, following the same approach previously applied to the powder obtained from the dried ripe coffee cherry pulp infusion [2]. The expected final product amount was estimated using the infusion’s concentration (°Brix), measured with a PR-32α digital refractometer (ATAGO, Tokyo, Japan), and the total weight (g) of the infusion volume subjected to drying.

$$\mathrm{Theoretical}\mathrm{value}\;(g\mathrm{of}\mathrm{expected}\mathrm{soluble}\mathrm{powder})\;\;\;=\frac{\mathrm{Concentration}\mathrm{of}\mathrm{infusion}\;\left({}^{\circ}\mathrm{Brix}\right)\times \mathrm{weight}\mathrm{of}\mathrm{infusion}\mathrm{to}\mathrm{dry}\left(g\right)}{100}$$

$$\mathrm{Yield}\mathrm{of}\mathrm{the}\mathrm{drying}\mathrm{process}\;(\%)=\frac{\mathrm{experimental}\mathrm{value}\;(g\mathrm{of}\mathrm{soluble}\mathrm{powder})\times 100}{g\mathrm{of}\mathrm{expected}\mathrm{soluble}\mathrm{powder}}$$

### 2.3. Phytochemical Characterization (Phenolics and Methylxanthines by HPLC-QTOF)

Phenolic compounds in Instant Cascara 1.0 and 2.0 were identified using an Agilent 1200 HPLC system (Santa Clara, CA, USA) equipped with a coupled degasser (G1322A), thermostated column module (G1316A), quaternary pump (G1311A), thermostated automated injector (G1367B), and diode array detector (G1315B). This setup was connected to a mass spectrometer (Agilent G6530A Accurate Mass QTOF LC/MS) (Agilent Technologies, Santa Clara, CA, USA) with an electrospray ionization (ESI) source incorporating JetStream technology. The system was operated using MassHunter Data Acquisition software (version B.05.00) and analyzed with MassHunter Qualitative Analysis (version B.07.00). Two µL of samples were injected in a ZORBAX Eclipse XDB-C18 column (150 mm × 4.6 mm × 5 µm) at 40 °C. Samples were diluted 1:10 before injection. The solvent gradient consisted of 0.1% formic acid (solvent A) and 0.1% formic acid in acetonitrile (solvent B), with the following elution profile (time, % solvent A): 0 min, 95%; 20 min, 85%; 30 min, 70%; 35 min, 50%; 37 min, 95%; and 45 min, 95%.

Methylxanthines in IC 1.0 and IC 2.0 were identified using an Agilent 1200 HPLC system (Santa Clara, CA, USA) as described above. Twenty µL of samples for theobromine and theophylline and 1 µL of sample for caffeine were injected in a ZORBAX Eclipse XDB-C18 column (150 mm × 4.6 mm × 5 µm) at 40 °C. Samples were diluted 1:10 before injection. The solvent gradient consisted of 0.1% formic acid (solvent A) and 0.1% formic acid in acetonitrile (solvent B), with the following elution profile (time, % solvent A): 0 min, 95%; 20 min, 85%; 30 min, 70%; 35 min, 50%; 37 min, 95%; and 45 min, 95%.

These methods are consistent with established approaches for the determination of phenolic compounds and methylxanthines in plant-based matrices. Phytochemical analyses were conducted in technical triplicate for each sample. Compounds not detected under the analytical conditions are reported as N.D. (not detected).

### 2.4. Cell Models and Experimental Conditions

Human cancer (HepG2 and Caco-2) and normal (CCD-18) cells, as well as murine macrophages (RAW 264.7), were cultured in DMEM supplemented with 10% heat-inactivated FBS, 50 U/mL penicillin, 50 µg/mL streptomycin and 50 µg/mL L-glutamine. Cells were incubated in a 5% CO_2_ atmosphere at 37 °C in a BINDER CB 210 incubator (BINDER GmbH, Tuttlingen, Germany). Caco-2 cell line was provided by the Bioanalytical Techniques Unit (Instituto de Investigación en Ciencias de la Alimentación, Consejo Superior de Investigaciones Científicas (CSIC)-Universidad Autónoma de Madrid (UAM), Madrid, Spain). CCD-18 and HepG2 cells were obtained from the Centro de Instrumentación Científica (CIC) of the Universidad de Granada, Spain.

Concentration ranges were selected based on cytotoxicity screening assays, assay-specific sensitivity requirements, and previously reported effective concentrations in similar in vitro models (Supplementary Figures S1–S3, [2]).

### 2.5. Antioxidant Potential: Intracellular ROS in Colon Cells

Caco-2 (human colorectal adenocarcinoma) and CCD-18 (human normal colon tissue) cells were seeded in 96-well plates (5 × 10^4^ and 1 × 10^4^ cells/well, respectively). After 24 h, the cells were treated with IC 1.0 and IC 2.0 at 100 µg/mL, based on our previous results [2]. Intracellular Reactive Oxygen Species (ROS) production was evaluated using DCFH-DA. After the incubation, 2 µL/well of DCFH-DA (5 mg/mL in DMSO) was added and the plates were incubated for 30 min at 37 °C, in the dark. Cells were washed once with 1X phosphate-buffered saline (PBS) and samples (100 µg/mL) were added for 45 min. Fluorescence intensity was measured in a fluorimeter microplate reader (CYTATION 5 microplate spectrophotometer, BioTek Instruments, Winooski, VT, USA) at 485 nm/528 nm (excitation/emission). Tert-butyl hydroperoxide (1 mM) was used as a positive oxidation control and vitamin C (10 µg/mL) as an antioxidant control. The negative control was the culture medium. To correct ROS values, an MTT assay for cell viability was performed on the same plate [14].

### 2.6. Anti-Inflammatory Potential in Macrophages (NO Production)

The anti-inflammatory effects of IC 1.0 and IC 2.0 were assessed in RAW 264.7 murine macrophages by quantifying NO production after LPS stimulation, using the Griess reaction [15]. Briefly, cells were cultured in 96-well plates (8 × 10^4^ cells/well), and pre-incubated with the samples (0.1 mg/mL) for 24 h. They were then co-treated with LPS (1 μg/mL) and the same concentration of samples for an additional 24 h. Supernatants were collected and mixed with Griess reagent (1% (*w*/*v*) sulphanilamide and 0.1% *w*/*v* n-1-(naphthyl)ethylenediaminedihydrochloride in 2.5% *v*/*v* H_3_PO_4_), and absorbance was measured at 550 nm. Untreated cells served as the control.

### 2.7. Genotoxicity Assessment by Comet Assay

The alkaline comet assay was performed according to the protocol described by Olive et al. (1992), with minor variations [16]. HepG2 cells (human hepatoma) were plated in a 24-well plate (1.5 × 10^5^ cells/well) for 24 h. Then, cells were treated with IC 0.0 (FD-IC), IC 0.0 (SD-IC), IC 1.0 and IC 2.0 at 10, 100 and 1000 µg/mL. After 24 h, cells (12 µL of a suspension of 1.5 × 10^5^ cells) were mixed with 70 µL of LMP agarose type VII (0.75% in PBS), placed on slides that had been pre-coated with LMP agarose type VII (0.30% in PBS), and incubated on ice. After drying, HepG2 cells were lysed in the dark in a high salt alkaline buffer (2.5 M NaCl, 0.1 M EDTA, 0.01 M Tris, 1% Triton X-100, pH 10) for 1 h. Subsequently, the slides were dipped in electrophoresis buffer (0.3 M NaOH, 1 mM EDTA, pH 13, cooled in a refrigerator) for 40 min in darkness, followed by electrophoresis (for 30 min at 25 V, in darkness). The slides were neutralized (0.4 M Tris pH 7.5), fixed in methanol and stained with 1× GelRed® in Tris-acetate EDTA (1X TAE) for 5 min. Lastly, cells were analyzed with a fluorescence microscope (OLYMPUS BH-2) using an image analysis system (Komet 5.1). Results were expressed as a percentage of Tail DNA and images of 50 randomly selected cells were processed for each concentration. BaP 100 µM, dissolved in sterile DMSO, was used as a positive control of DNA damage and culture medium as a negative control.

### 2.8. Chemoprotective Effect Against BaP-Induced DNA Damage

The comet assay was used to determine the protective effect of the samples against BaP. HepG2 cells were seeded onto 24-well plates (1.5 × 10^5^ cells/well). Twenty-four hours after plating, IC 0.0 (FD-IC), IC 0.0 (SD-IC), IC 1.0 and IC 2.0 (1, 10 and 100 µg/mL) were added to the wells, and the plates were incubated for 24 h. Then, cells were co-treated with BaP (100 µM) and the concentrations of samples previously indicated, for another 24 h. After the co-administration of BaP and samples, the cells were prepared and analyzed as described in the previous Section 2.7.

### 2.9. Antiproliferative Potential in Normal and Cancer Colon Cells

Colony formation assay was carried out to study cell proliferation in normal and cancerous colon cells. Cells were seeded in 24-well plates (Caco-2: 1 × 10^4^ cells/well, CCD-18: 2.5 × 10^4^ cells/well). After 24 h, cells were treated with all Instant Cascara formulations (IC 0.0 (FD-IC), IC 0.0 (SD-IC), IC 1.0 and IC 2.0) at 100 µg/mL and incubated for 7 days. The cell colonies were washed with 1× PBS and fixed with 3% paraformaldehyde for 10 min. Then, they were permeabilized with 2% methanol for 2 min. Finally, cells were stained with 0.5% crystal violet in 20% methanol for 10 min, washed twice with H_2_O and left to dry. Microscopy images were acquired using a Leica DMi1 optical microscope (Leica Biosystems, Barcelona, Spain) at defined magnifications (×10), and representative fields were selected from independent experiments. Scale bars were included for reference using ImageJ software (version 1.53t, NIH, Bethesda, MD, USA) [17]. In addition, the wells were destained with 0.1 M sodium citrate in 50% ethanol (pH 4.2), and absorbance was measured at 550 nm using a BioTek Epoch 2 Microplate Spectrophotometer (Winooski, VT, USA). Control of normal cell proliferation was culture medium.

### 2.10. Cell Cycle Modulation and Apoptosis Induction

The effect of IC 0.0 (FD-IC), IC 0.0 (SD-IC), IC 1.0 and IC 2.0 (0.5–2 mg/mL) on the cell cycle was assessed using flow cytometry, as previously described by Sánchez–Martín et al. [18]. Caco-2 cells (0.5 × 10^6^ cells/well) were seeded in a 6-well plate. After treatment of cells with different concentrations of samples for 24 h, cells were collected, washed twice with 1X PBS and fixed in 70% ethanol. DNA was stained with 50 μg/mL PI and RNase A (1 mg/mL) was added, for 30 min at 37 °C in a dark environment. Finally, fluorescence was measured using a FACS Calibur flow cytometer (Beckton Dickinson, Franklin Lakes, NJ, USA). Flowing Software 2.5.1 was used to analyze the results. Data were expressed as the percentage of cells in the different phases (SubG1, G0/G1, S and G2/M) over the total cells. Etoposide (300 µM) was used as a positive control for changes in cell cycle phases and culture medium as a negative control.

eBioscience™ Annexin V-FITC Apoptosis Detection Kit was employed to identify cell death by apoptosis. Caco-2 cells were seeded in 6-well plates (0.5 × 10^6^ cells/well) and were treated with all Instant Cascara formulations (IC 0.0 (FD-IC), IC 0.0 (SD-IC), IC 1.0 and IC 2.0) at concentrations between 0.5 and 2 mg/mL, for 24 h. Briefly, cells were resuspended in 100 µL 1X annexin binding buffer and incubated with 5 µL of annexin V-FITC for 10 min. They were washed once with 1X annexin binding buffer, and 5 µL of PI was added. The samples were then kept on ice until analysis. Fluorescence was measured using a FACSCalibur flow cytometer (Beckton Dickinson, Franklin Lakes, NJ, USA) from the Flow Cytometry and Fluorescence Microscopy Unit of the Universidad Complutense de Madrid (UCM). The Flowing Software 2.5.1 (Perttu Terho, Turku Center for Biotechnology, University of Turku, Turku, Finland) was used for the evaluation of the results. Etoposide (300 µM) was used as a positive control of apoptosis. The negative control was the culture medium.

### 2.11. Receptor-Mediated Responses (Tongue/Gut-on-a-Chip Assays)

These receptor-based assays are designed to assess direct ligand–receptor interactions and do not require gastrointestinal digestion, as they do not aim to reproduce physiological bioavailability but to characterize formulation-dependent interaction patterns at the receptor level.

Sensory- and neurotransmitter-related receptor responses were evaluated using validated in vitro cell-based platforms. These provide mechanistic clues of potential sensory-neuroactive compositions on specific tested receptor sets, but as such do not yet predict the full complexity of human sensory perception. Any (taste) bioactivity or modulation should therefore be confirmed in sensory panel evaluations. Both a fluorescence [19] and bioluminescence-based microfluidic tongue/gut-on-a-chip receptomics assay was employed to obtain direct quantitative data on the activation of multiple bitter, sweet, chemesthetic and neurotransmitter receptors in response to the injection of complex cascara samples and controls. The complete list of receptors and controls is detailed in the supplemental data (Supplementary Tables S1 and S2). Each receptor was present with 10 technical replicas on the array.

The two coffee cascara beverages were prepared by dissolving 10 mg/mL IC 0.0 (SD-IC) and IC 2.0, based on our previous study [2], directly in assay buffer resembling a consumer concentration. This yielded deep red colored extracts which were sequentially injected at 1 and 10 mg/mL extract on a fluorescence-based receptor assay with an array containing neurotransmitter GPCR’s and a Förster resonance energy transfer (FRET) calcium probe, as described by Roelse [19]. The 10 mg/mL extract showed such high sample autofluorescence that no receptor-specific analysis was possible, but at 1 mg/mL the extract could be analyzed.

The high sample autofluorescence prevented measurements at the more taste-relevant concentrations (10 mg/mL) [2]. Therefore, a novel bioluminescence resonance energy transfer (BRET) analysis was performed using the calcium probe Calflux VTN (Addgene #83926, Carl Johnson lab, Nashville, TN, USA), composed of the Nanoluc luciferase and a Venus YFP domain connected by a Troponin calcium-binding domain [20]. A panel of 13 commonly active bitter taste receptors, including two haplotypes of R38, the sweet receptor TAS1R2/3, the satiety receptor GLP-1, the serotonin receptor HTR2C, and the muscarinic M3 receptor, were tested against sequential injections of the extracts at 10 mg/mL. The bioluminescence assay depends on the activation of Nanoluc by fumarizine (Nano-Glo® Live Cell Assay System, Promega, Madison, WI, USA) substrate to convert into light. Therefore, the microfluidic assay provides a constant and stable flow of substrate mixed into the assay flow. During the bioluminescence measurements, the samples were diluted with bioluminescence substrate for NanoLuc and this resulted in a final 9 mg/mL sample concentration.

In both FRET and BRET assays, the ratio response, as detected with the emission filters Em 480/40 and 535/30, represented the change in calcium concentration. The figures show smoothed, raw ratio traces of the blank, a positive control (ATP) and the extract samples in sequential injections. The assay buffer flow during the assay washes the samples from the array, which is verified by a return to baseline of the ratio traces.

### 2.12. Statistical Analysis

All cell-based assays were performed in three independent biological replicates, each including technical replicates. Phytochemical analyses were conducted in technical triplicate for each sample. Results are expressed as mean ± standard deviation (SD).

Receptor-based assays were performed using multiple technical replicates (*n* = 10 spots per condition), as defined by the platform design.

Statistical comparisons were carried out using Student’s *t*-test or one-way ANOVA, followed by Tukey’s post hoc test, depending on the experimental design. Analyses were performed using Statgraphics Centurion 19 software (Statgraphics Technologies, Inc., The Plains, VA, USA), and *p* < 0.05 was considered statistically significant.

Statistical analyses were conducted independently for each experimental endpoint, with post hoc tests applied within each assay. No correction for multiple comparisons across different assays was applied.

## 3. Results and Discussion

### 3.1. Yield and Technological Performance of Spray-Dried Instant Cascara

Carrier incorporation during spray-drying markedly improved process yield, confirming its technological relevance for cascara-based products. Compared to carrier-free spray-drying, which achieved yields below 25%, the addition of inulin-based carriers increased recovery up to 76.6% for HPX and 56.0% for Synergy1. HPX, composed of long-chain inulin, provided the highest efficiency, while Synergy1, enriched with oligofructose, showed lower performance likely due to its higher hygroscopicity. These results are consistent with previous reports on the role of polymer chain length in forming stable matrices during drying [21], and highlight the importance of carrier selection for process optimization. While higher yields were expected due to the addition of a spray-dryable carbohydrate, the scientific interest of this study lies in evaluating whether carrier incorporation also modulates the functional properties of Instant Cascara beyond process efficiency.

From an industrial perspective, improving yield and powder stability reduces production costs and facilitates the development of cascara-based ingredients with standardized quality.

### 3.2. Influence of Carrier Polymerization Degree on Bioactive Profile and Functional Responses

Beyond their effect on drying performance, inulin-type carriers with different degrees of polymerization can modify the physical properties of the cascara matrix, influencing the measurable recovery or apparent stability of its native bioactive compounds, including their retention, protection, or release during analysis.

This section examines the phytochemical composition of the carrier-containing powders and evaluates biological responses inherent to the cascara matrix, including antioxidant capacity, anti-inflammatory activity, chemoprotection, modulation of cell proliferation, and apoptosis induction.

#### 3.2.1. Phytochemical Composition and Phenolic Profile

Instant Cascara powders (IC 1.0 and IC 2.0) bioactive profiles are shown in Table 2. Significant differences (*p* < 0.05) were observed for caffeine, catechin dimer, and 5-O-feruloylquinic acid, all of which were lower in IC 2.0 compared to IC 1.0.

For other methylxanthines (theobromine and theophylline) and most phenolic compounds, including chlorogenic acids, mangiferin, and rutin, no significant differences were detected between the two formulations (*p* > 0.05). Anthocyanins were not detected in either sample, confirming previous observations that these compounds are not retained during spray-drying of cascara infusion, although they may appear after freeze-drying [2].

New inulin-containing formulations showed lower values of total methylxanthines (Table 2) than those previously found in IC 0.0 obtained by spray-drying [2]. Catechin derivatives were detected only in carrier-containing samples, indicating possible stabilization of these flavonoids within the inulin matrix. Conversely, mangiferin and rutin were more abundant in IC 0.0 (SD-IC), which could reflect stronger interactions of these compounds with inulin, reducing their extractability [22]. Such behavior is consistent with previous observations showing that the incorporation of inulin-type carriers may lead to an apparent reduction in analytical recovery of phenolic compounds, which can be explained by a combination of solid dilution effects and matrix–polyphenol interactions, including physical entrapment or encapsulation during spray-drying [23].

For hydroxycinnamic acids, both IC 1.0 and IC 2.0 retained major chlorogenic acids and feruloyl derivatives, with IC 1.0 showing slightly higher values overall (e.g., 3-O-caffeoylquinic acid: 4933.26 ± 279.35 µg/g in IC 1.0 vs. 4756.53 ± 145.49 µg/g in IC 2.0). Although the total phenolic content was lower in carrier-containing samples (15.19 mg/g for IC 1.0 and 13.08 mg/g for IC 2.0) than in IC 0.0 (SD-IC) [2], the overall phenolic profile remained similar. Previous studies have suggested that encapsulation may influence analytical recovery and compound accessibility during digestion, although these aspects were not evaluated in the present study [22,24,25].

#### 3.2.2. Antioxidant Activity (ROS Reduction in Colon Cells)

To evaluate whether carrier incorporation impacts antioxidant functionality, intracellular ROS levels were measured in normal and cancerous colon cells. Both formulations, IC 1.0 and IC 2.0, demonstrated significant ROS reduction, indicating preserved antioxidant potential despite differences in phenolic retention.

**Figure 2:**
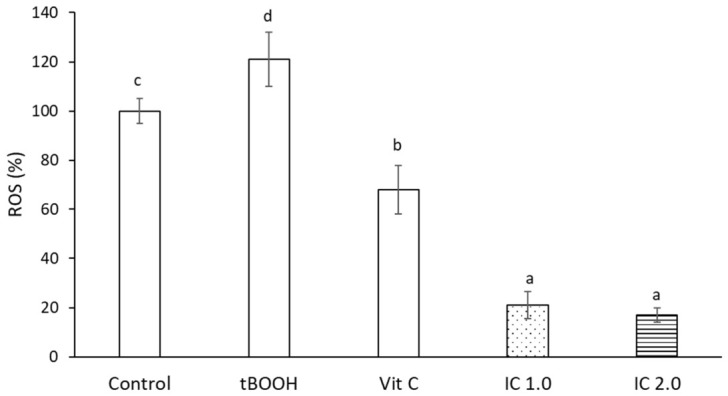
Basal intracellular ROS formation in CCD-18 cells treated with IC 1.0 and IC 2.0 (0.1 mg/mL). Cells without treatment were considered as a physiological control; tBOOH (1 mM) and vitamin C (0.1 mg/mL) were used as an oxidant and antioxidant control, respectively. Data are expressed as mean ± SD (*n* = 3). Different letters indicate significant differences (Tukey’s test; *p* < 0.05).

Oxidative stress can disrupt normal cellular structures and functions, contributing to pathological conditions such as inflammation, cancer and neurodegenerative diseases [26]. Intracellular ROS levels were therefore assessed in human colon cancer (Caco-2) and normal colon (CCD-18) cells at 0.1 mg/mL, a concentration selected based on MTT cytotoxicity results (Supplementary Figures S1 and S2) and previous work [2]. In CCD-18 cells (Figure 2), the oxidant agent tBOOH significantly increased intracellular ROS production, whereas vitamin C significantly reduced it (*p* < 0.05). Both IC 1.0 and 2.0 also decreased ROS levels (*p* < 0.05), with no significant differences observed between them.

**Figure 3:**
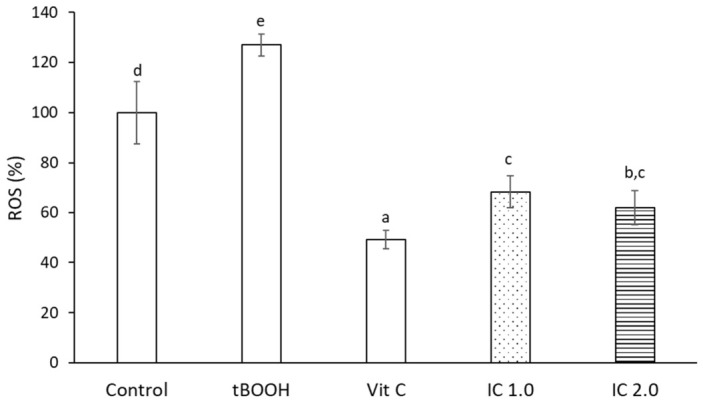
Effect of IC 1.0 and IC 2.0 (0.1 mg/mL) on basal intracellular ROS formation in Caco-2 cells. Untreated cells were considered a physiological control, tBOOH (1 mM) was used as an oxidation control, and vitamin C (0.1 mg/mL) was used as an antioxidant control. Data are expressed as mean ± SD (*n* = 3). Different letters indicate significant differences (Tukey’s test; *p* < 0.05).

Basal ROS production in Caco-2 cells is shown in Figure 3. The oxidant control (tBOOH) significantly (*p* < 0.05) increased ROS formation, whereas vitamin C decreased it. Both IC 1.0 and 2.0 reduced ROS levels (*p* < 0.05), with no significant differences observed between them.

Maintaining balanced ROS levels in colon cells is relevant for studying oxidative stress-related processes. Indeed, correlations have been reported between the total antioxidant capacity of foods and reduced colorectal cancer risk [27], while cancer progression has been associated with oxidative stress, inflammation, and DNA damage [28]. Cancer cells typically exhibit elevated ROS levels compared to normal cells [29]. In this context, the observed effects in Caco-2 cells suggest that IC formulations modulate ROS levels under the in vitro conditions tested. Castaldo et al. reported that coffee brews at 0.25–0.50 mg/mL reduced ROS by approximately 20% in HT-29 colorectal adenocarcinoma cells after 24 h [30], whereas Colombian coffee at 500 µg/mL did not affect basal ROS levels in Caco-2 cells [31].

Endogenous and exogenous antioxidants, particularly phenolic compounds, play a role in counteracting ROS [32]. Both IC 0.0 (FD-IC) and IC 0.0 (SD-IC), contain major phenolic compounds such as mangiferin, caffeoylquinic acid, chlorogenic acid, and 5-O-feruloylquinic acid [2]. Mangiferin has been shown to enhance antioxidant enzymes in a rat model of induced colorectal cancer [33], while caffeoylquinic and chlorogenic acids decreased intracellular ROS in Caco-2 cells [34]. The addition of inulin to IC 0.0 (SD-IC) did not further improve antioxidant effects. However, inulin may exert indirect ROS-scavenging activity via short-chain fatty acids produced during colonic fermentation and by stimulating antioxidant enzymes [35].

#### 3.2.3. Anti-Inflammatory Potential (NO Inhibition in Macrophages)

The ability of cascara formulations to modulate inflammatory responses was assessed through NO production in LPS-stimulated macrophages. Results revealed carrier-dependent differences, highlighting the role of structural composition in anti-inflammatory efficacy.

**Figure 4:**
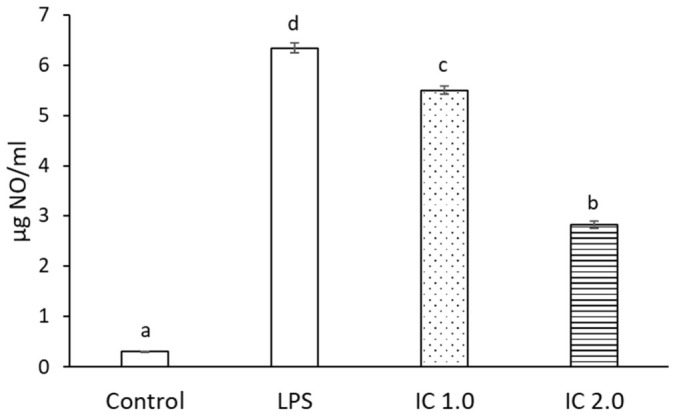
Effect of IC 1.0 and IC 2.0 on nitric oxide formation in RAW 264.7 macrophages. The control condition corresponded to untreated cells, while lipopolysaccharide (LPS, 1 μg/mL) was applied as the pro-inflammatory stimulus. Cells were pre-exposed to the samples (0.1 mg/mL) for 24 h, followed by a simultaneous treatment with LPS and the corresponding sample for an additional 24 h. Data are expressed as mean ± SD (*n* = 3). Different letters indicate significant differences (Tukey’s test; *p* < 0.05).

Figure 4 illustrates the effect of IC 1.0 and IC 2.0 (0.1 mg/mL) on NO production in RAW 264.7 macrophages (Supplementary Figure S3). As expected, LPS (1 μg/mL) significantly increased NO production compared to untreated control cells, while both samples significantly reduced LPS-induced NO levels (*p* < 0.05). Notably, IC 2.0 showed a significantly stronger inhibitory effect than IC 1.0 (*p* < 0.05).

The stronger inhibition observed for IC 2.0 may be related to differences in structural composition. While HPX consists primarily of high-molecular-weight inulin, Synergy1 combines long-chain inulin with shorter oligofructose fractions, which may influence the interaction of bioactive components within the matrix.

These findings indicate that both inulin-enriched IC beverages attenuate LPS-induced NO production in macrophages, supporting in vitro anti-inflammatory activity under the conditions tested.

Chronic inflammation is a major contributor to the onset and progression of non-communicable diseases such as cardiovascular and neurodegenerative diseases, type 2 diabetes, and cancer [36]. Nitric oxide, overproduced by activated macrophages via inducible nitric oxide synthase (iNOS), plays a central role in this process. Sustained NO release amplifies inflammatory signaling and contributes to oxidative stress and tissue damage [37].

In this context, the ability of IC beverages to attenuate LPS-induced NO production highlights their potential relevance in modulating inflammatory responses under the in vitro conditions tested. These results are consistent with our previous work [2], where IC 0.0 (FD-IC) and IC 0.0 (SD-IC) demonstrated antioxidant and anti-inflammatory activity. Here, we extend those observations by showing that inulin-enriched formulations, particularly IC 2.0, could downregulate a canonical macrophage inflammatory response.

This effect may be associated with formulation-dependent matrix interactions influencing the availability of cascara bioactive compounds, although no direct assessment of bioavailability was performed. For example, mangiferin has been reported to inhibit inflammatory gene expression and regulate cytokine production and macrophage activation [38].

The slightly stronger response observed for IC 2.0 may relate to differences in matrix interactions associated with its lower degree of polymerization, possibly affecting the availability or stability of cascara bioactive compounds within the cellular environment.

New products containing inulin-type carriers (IC 1.0 and IC 2.0) showed differential antioxidant and anti-inflammatory responses, properties previously ascribed to IC 0.0 [2]. These observations are consistent with broader evidence indicating that dietary components can modulate oxidative stress and inflammatory processes through multiple interconnected mechanisms [39].

#### 3.2.4. Genotoxicity and Chemoprotective Effects

Safety assessment is critical for novel food ingredients. Comet assays confirmed the absence of genotoxicity in all cascara formulations and demonstrated protective effects against BaP-induced DNA damage under the experimental conditions. Their findings support a preliminary safety profile at the in vitro level.

HepG2 cells are widely used for genotoxicity assessment, showing high accuracy in in vitro comet assays [40,41]. Therefore, the genotoxicity of Instant Cascara beverages was examined using this model.

**Figure 5:**
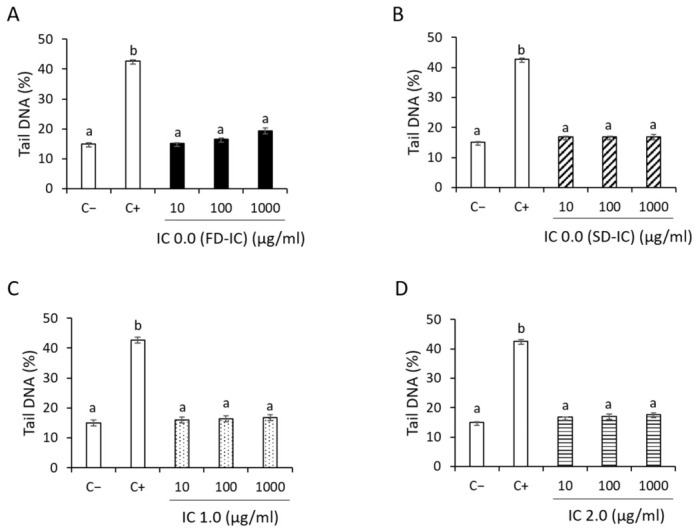
Genotoxicity effect of IC samples evaluated by comet assay on HepG2 cells. Cells treated with and without the addition of BaP (100 µM) were used as controls (C+ and C−, respectively). Panels (**A**–**D**) show the dose-dependent effect of a single addition of IC 0.0 (FD-IC), IC 0.0 (SD-IC), IC 1.0 and IC 2.0, respectively (10, 100 and 1000 µg/mL). Data are expressed as mean ± SD (*n* = 3). Different letters indicate significant differences (Tukey’s test; *p* < 0.05).

Figure 5 depicts the genotoxic response of IC 0.0 (FD-IC), IC 0.0 (SD-IC), IC 1.0 and IC 2.0 at concentrations of 10, 100 and 1000 µg/mL in hepatic cells. None of the tested samples or concentrations showed genotoxicity, as no statistically significant differences (*p* > 0.05) were detected compared with the negative control. In contrast, significant differences (*p* < 0.05) were observed only with the positive control, benzo(a)pyrene (BaP, 100 µM). BaP is a polycyclic aromatic hydrocarbon commonly found in smoke and thermally processed foods. It is classified as a Group 1 human carcinogen by the International Agency for Research on Cancer (IARC), and its detoxification has been linked to cytochrome P450 (CYP450) activity [42].

Altogether, these findings support the food safety of the IC beverages evaluated under the conditions tested.

**Figure 6:**
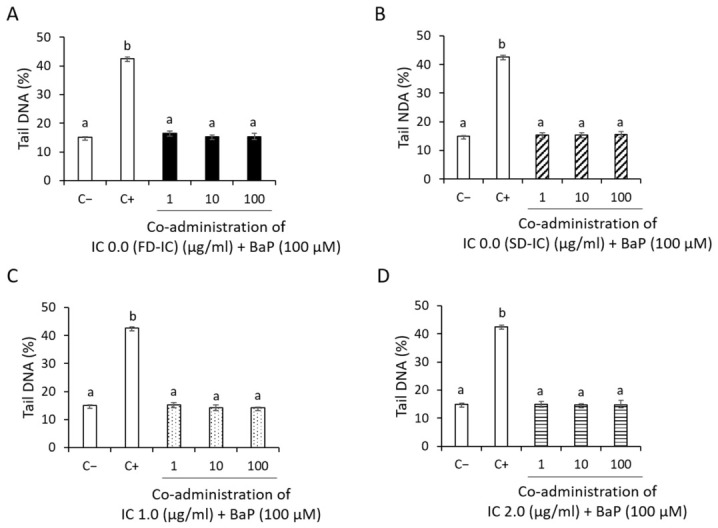
Chemoprotective effect against BaP of IC samples on HepG2 cells. Cells treated with and without the addition of BaP (100 µM) were used as controls (C+ and C−, respectively). (**A**) cells treated with BaP (100 µM) and IC 0.0 (FD-IC) (1, 10 and 100 µg/mL). (**B**) cells treated with BaP (100 µM) and IC 0.0 (SD-IC) (1, 10 and 100 µg/mL). (**C**) cells treated with BaP (100 µM) and IC 1.0 (1, 10 and 100 µg/mL). (**D**) cells treated with BaP (100 µM) and IC 2.0 (1, 10 and 100 µg/mL). Data are expressed as mean ± SD (*n* = 3).

We also investigated the chemoprotective effect of IC samples against BaP-induced DNA damage (Figure 6). Co-treatment with IC 0.0 (FD-IC), IC 0.0 (SD-IC), IC 1.0 and IC 2.0 (1–100 µg/mL) alongside BaP (100 µM) reduced the DNA damage, with tail lengths approaching those of the negative control. These protective effects are consistent with the known properties of cascara-derived phenolics. Carrier addition did not introduce new biological activities but preserved those naturally present in Instant Cascara.

These results suggest an in vitro protective effect of IC beverages against this mutagen in liver cells. Similar chemoprotective effects of coffee bioactive compounds have been reported against the cooked food mutagen 2-amino-1-methyl-6-phenylimidazo[4,5-b]-pyridine (PhIP), resulting in decreased DNA adduct formation in rats [43]. Additionally, coffee by-products have shown comparable effects against BaP in liver cells [44].

#### 3.2.5. Antiproliferative Activity in Colon Cancer Cells

The impact of carrier structure on cell proliferation was examined in normal and cancerous colon cells. All formulations selectively inhibited tumor cell growth without affecting normal cells, highlighting their relevance for exploring mechanisms associated with colorectal cancer in in vitro models.

**Figure 7:**
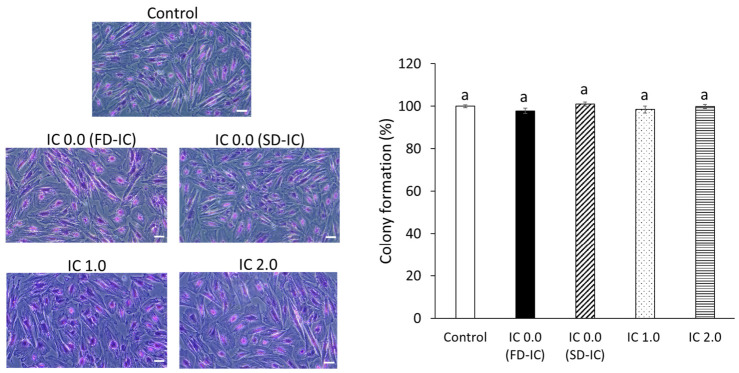
Effect of IC samples (0.1 mg/mL) on the proliferation of CCD-18 cells. Representative images of crystal violet-stained cultures obtained by light microscopy (magnification ×10; scale bar = 200 µm), are shown in the (**left**) panel. Images correspond to representative fields from independent experiments. The (**right**) panel shows the colony formation (%) as the mean of absorbance. Data are expressed as mean ± SD (*n* = 3). Same letters indicate no significant differences (Tukey’s test; *p* > 0.05).

**Figure 8:**
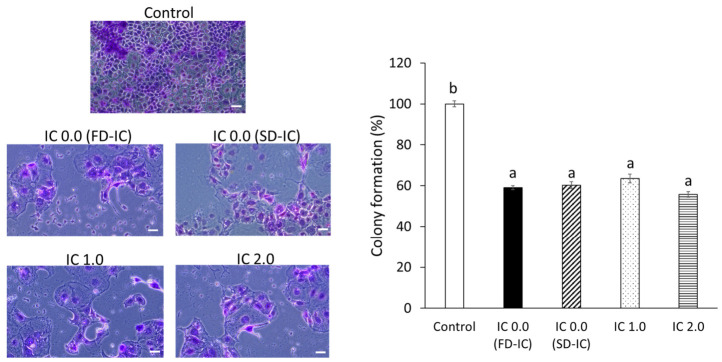
Effect of IC samples (0.1 mg/mL) on the proliferation of Caco-2 cell line. (**Left**) panel: representative images of crystal violet-stained cultures obtained by light microscopy (magnification ×10; scale bar = 200 µm). Images correspond to representative fields from independent experiments. (**Right**) panel: bar graph of colony formation percentage (absorbance). Data are expressed as mean ± SD (*n* = 3). Different letters indicate significant differences (Tukey’s test; *p* < 0.05).

Cell proliferation is tightly regulated in multicellular organisms, whereas uncontrolled proliferation is a hallmark of cancer [45]. In this study, the effect of IC beverages on the proliferation of human normal and cancer colon cells was assessed (Figure 7 and Figure 8). As shown in Figure 7, none of the beverages affected the proliferation of normal colon cells. In contrast, all samples significantly (*p* < 0.05) reduced tumor cell proliferation (Figure 8), with reduction percentages of 41.02%, 39.77%, 36.46% and 44.42% for IC 0.0 FD-IC, IC 0.0 (SD-IC), IC 1.0 and IC 2.0, respectively, at 0.1 mg/mL. No significant differences were found among the formulations.

Pharmacological cancer therapies are often limited by their toxicity to normal cells [46]. The selective effect observed here, where IC formulations reduced proliferation only in tumor cells, highlights a potential advantage. Similar results were reported by Bułdak et al., who described that kahweol, a diterpene present in coffee, inhibited the proliferation of colorectal cancer cells without affecting normal colon cells (CCD-18) [47].

Caffeine, present in IC beverages [2], and chlorogenic acid [48], have been shown to decrease proliferation in colorectal cancer cells, in agreement with our results.

In multicellular organisms, cell proliferation is tightly regulated to maintain tissue homeostasis, with intrinsic limits on proliferative capacity. Disruptions of these regulatory pathways, such as uncontrolled proliferation or stress-induced signaling, can lead to the accumulation of ROS, which act as both signaling molecules and mediators of cellular damage. When ROS levels exceed the physiological thresholds, apoptosis can be triggered, serving as a safeguard against aberrant proliferation and oncogenic transformation [45].

#### 3.2.6. Cell Cycle Modulation and Apoptosis Induction

To further explore antiproliferative mechanisms, cell cycle distribution and apoptosis were analyzed. Carrier type influenced apoptotic response, with Synergy1 showing stronger effects than HPX, suggesting that formulation modulates bioactive compound functionality.

Apoptosis, a programmed form of cell death, plays a central role in maintaining tissue homeostasis. Tumor cells often evade apoptosis to sustain uncontrolled proliferation; therefore, induction of apoptosis represents a key mechanism targeted by anti-tumor therapies. In addition, alterations in genes regulating the cell cycle can disrupt the normal control of cell growth and division, driving cancer progression [49]. Consequently, we assessed whether IC beverages could alter cell cycle distribution and induce apoptosis.

**Figure 9:**
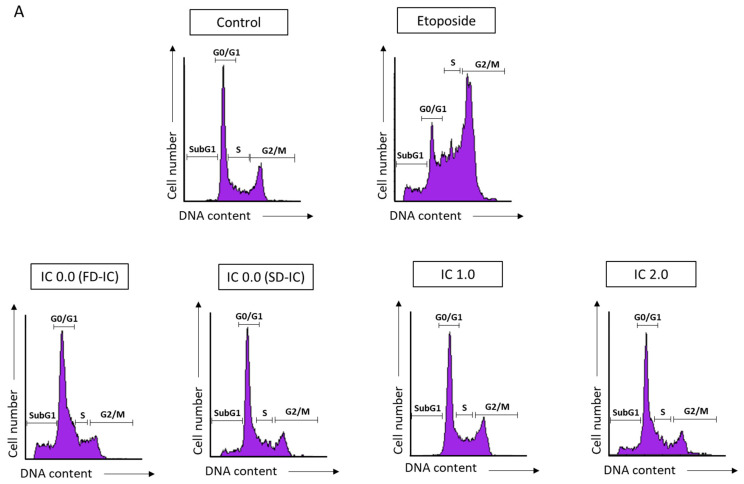

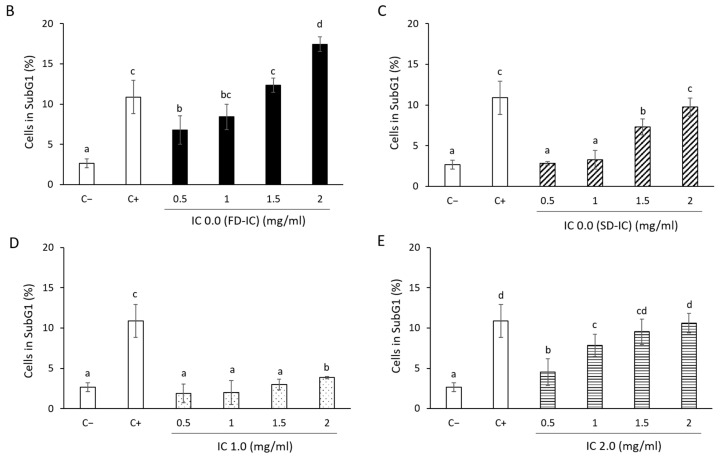
Effect of IC samples on cell cycle distribution in Caco-2 cells after 24 h of treatment. (**A**) Representative histograms of cell cycle phases obtained by flow cytometry after treatment with instant cascara (IC) beverages at 2 mg/mL. Peaks corresponding to SubG1, G0/G1, S, and G2/M phases are indicated. The control (untreated) and etoposide at 300 µM (positive control) profiles are included for comparison. (**B**–**E**) Quantification of the SubG1 population (apoptotic cells) in Caco-2 cells treated with (**B**) IC 0.0 (FD-IC), (**C**) IC 0.0 (SD-IC), (**D**) IC 1.0, and (**E**) IC 2.0 at concentrations ranging from 0.5 to 2 mg/mL. The histograms correspond to representative results from independent biological replicates. Data are expressed as mean ± SD (*n* = 3). Different letters indicate significant differences (Tukey’s test; *p* < 0.05).

The effect of Instant Cascara beverages on the cell cycle of Caco-2 cells was evaluated after 24 h of treatment at concentrations ranging from 0.5 to 2 mg/mL (Figure 9). All formulations produced a concentration-dependent increase in the SubG1 population (*p* < 0.05), consistent with apoptotic cell death (Figure 9B–E). Among the samples, IC 0.0 (FD-IC) had the strongest response, with SubG1 values rising from 6.77% at 0.5 mg/mL to 17.46% at 2 mg/mL, exceeding the apoptotic levels induced by the other formulations. IC 2.0 also produced a marked increase, reaching 10.59% SubG1 at 2 mg/mL, whereas IC 0.0 (SD-IC) exhibited a moderate effect (9.74% at 2 mg/mL), and IC 1.0 only minor changes. These results confirm that the apoptotic effect depends on both concentration and formulation, being more pronounced in IC 0.0 (FD-IC), IC 2.0 and IC 0.0 (SD-IC).

As expected, etoposide markedly increased both the SubG1 fraction (10.89%) and the proportion of cells in G2/M (Figure 9A), confirming its role as a positive control [50]. This contrasts with the response observed for the IC beverages, which triggered apoptosis without altering the distribution of cells in G0/G1, S, or G2/M phases. Similar patterns have been reported for chlorogenic acid, a major phenolic compound in cascara, which can induce cell cycle alterations depending on concentration [51].

Overall, the cell-cycle analysis showed a dose-dependent increase in the SubG1 fraction, particularly for IC 0.0 (FD-IC), IC 0.0 (SD-IC), and IC 2.0. This pattern is consistent with apoptosis rather than cell-cycle arrest and was further confirmed by the subsequent Annexin V assays.

**Figure 10:**
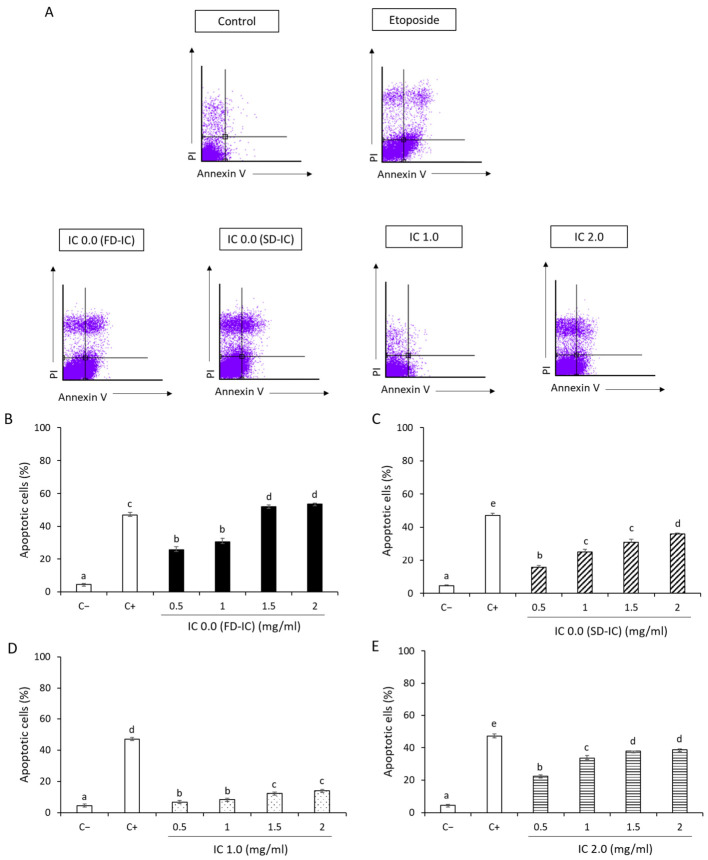
Effect of IC samples on apoptosis induction in Caco-2 cells after 24 h of treatment. (**A**) Representative dot plots of Annexin V/propidium iodide (PI) staining obtained by flow cytometry after treatment with instant cascara (IC) beverages at 2 mg/mL. Quadrants indicate: lower left (Annexin V^−^/PI^−^, viable cells), lower right (Annexin V^+^/PI^−^, early apoptotic cells), upper right (Annexin V^+^/PI^+^, late apoptotic cells), and upper left (Annexin V^−^/PI^+^, necrotic cells). The control (untreated) and etoposide at 300 µM (positive control) profiles are included for comparison. (**B**–**E**) Apoptotic cells percentage (early + late apoptosis) treated with (**B**) IC 0.0 (FD-IC), (**C**) IC 0.0 (SD-IC), (**D**) IC 1.0, and (**E**) IC 2.0 at concentrations ranging from 0.5 to 2 mg/mL. The dot plots correspond to representative results from independent biological replicates. Data are expressed as mean ± SD (*n* = 3). Different letters indicate significant differences (Tukey’s test; *p* < 0.05).

The pro-apoptotic effect of the Instant Cascara beverages was further evaluated by Annexin V–FITC/PI staining in Caco-2 cells after 24 h of treatment (Figure 10). Representative dot plots obtained at 2 mg/mL are shown in Figure 10A. As expected, etoposide (positive control) induced a clear shift toward both early (Annexin V^+^/PI^−^) and late apoptosis (Annexin V^+^/PI^+^). All cascara formulations also promoted apoptotic cell death, although with differences. IC 0.0 (FD-IC) displayed the most pronounced apoptotic profile, followed by IC 2.0, whereas IC 0.0 (SD-IC) showed a moderate response, and IC 1.0 only minor changes. These dot plots illustrate the total apoptotic fraction (early + late apoptosis) and correspond with the quantitative data presented in Figure 10B–E.

In Figure 10B–E, the positive control (etoposide) markedly increased total apoptosis (early + late) to 47.06%, confirming assay responsiveness. All IC formulations induced a concentration-dependent increase in apoptotic cells, although with clear differences. IC 0.0 (FD-IC) showed the strongest response, with total apoptosis rising from 25.79% at 0.5 mg/mL to 53.53% at 2 mg/mL (*p* < 0.05). IC 2.0 also produced a pronounced effect, reaching values close to 40% apoptosis at the highest doses (1.5 and 2 mg/mL). IC 0.0 (SD-IC) exhibited a more moderate increase (~30% and ~36% at 1.5 and 2 mg/mL, respectively), and IC 1.0 only minor changes (remaining below 14% total apoptosis even at 2 mg/mL).

These results confirm that the cascara beverages trigger apoptotic cell death in a dose-dependent manner, with IC 0.0 (FD-IC) and IC 2.0-enhanced formulations showing the highest efficacy. The observed effects are consistent with the contribution of bioactive constituents present in Instant Cascara. Carrier addition influenced the magnitude of these responses, suggesting an effect on the interaction of these compounds within the matrix.

The marked differences observed among formulations suggest that the apoptotic potential of the beverages is strongly influenced by processing conditions and matrix composition [52]. IC 0.0 (FD-IC), which displayed the highest values, likely retains greater levels of thermolabile phenolics related to apoptotic mechanisms due to the absence of heat exposure during freeze-drying [12,53]. The stronger response observed for IC 2.0 also supports the idea that inulin used as a carrier may influence the stability or release of phenolics, potentially facilitating their cellular uptake. These observations are consistent with previous reports indicating that extraction and processing methods substantially modulate the bioactivity of coffee-derived ingredients [12].

For clarity, a summary of the statistical comparisons among IC 0.0 (FD-IC), IC 0.0 (SD-IC), IC 1.0 and IC 2.0 is provided in Supplementary Table S3.

Taken together, these findings indicate that carrier addition affects phenolic retention and biological responses, reinforcing its multifunctional role in optimizing cascara-based ingredients.

From a formulation perspective, a trade-off between production yield and biological response can be considered. IC 1.0 showed a higher spray-drying yield, which is advantageous for industrial processing. In contrast, IC 2.0 exhibited stronger responses in selected in vitro assays, suggesting that different inulin structures may differentially modulate matrix interactions and the expression of bioactivity. Therefore, the selection of carrier type may depend on the targeted application, with priorities ranging from process efficiency to functional properties.

Based on the compositional data (Table 2), the nature of the carrier appears to influence the effective concentration of selected bioactive compounds within the formulation. Significant differences between IC 1.0 and IC 2.0 were observed for caffeine, catechin dimer, and 5-O-feruloylquinic acid, all of which have been associated with receptor-level activity, particularly in relation to bitter taste receptors.

From a receptor-based perspective, these results suggest that formulation-dependent differences might arise from variations in the availability of receptor-active ligands rather than from direct effects of the carrier itself. In this context, differences in receptor activation patterns can be interpreted as a function of the effective concentration and composition of bioactive compounds present in each formulation.

Importantly, caffeine is present at substantially higher concentrations than individual phenolic compounds (mg/g vs. µg/g range, Table 2), which implies a higher effective ligand availability under the experimental conditions. This supports the interpretation that caffeine is likely a dominant contributor to bitter receptor activation, while phenolic compounds may provide complementary contributions.

In addition to its role in bitter taste receptor activation, the observed modulation of M3 and D3/D4 receptor signaling is also consistent with the presence of caffeine and other bioactive compounds known to influence cholinergic and dopaminergic pathways, although no direct receptor agonism is implied in this receptor-based system.

Although the oligofructose-enriched carrier contains a fraction of free mono- and disaccharides, no activation of sweet taste receptors was observed under the experimental conditions, indicating that their contribution to receptor-level responses is likely negligible.

Inulin-type fructans are not considered direct ligands of taste or neurotransmitter receptors and are therefore unlikely to contribute directly to receptor activation. Their role is more plausibly associated with modulation of the formulation matrix, which may influence compound availability and interaction with receptor systems.

Beyond biological responses, carrier addition may influence sensory perception, as discussed in the following section.

### 3.3. Receptor-Mediated Responses (Sensory- and Neurotransmitter-Related)

The receptor-mediated responses obtained using in vitro platforms provide mechanistic insight into receptor-level interactions related to sensory- and neurotransmitter-associated targets of Instant Cascara on the tongue or in the gut. These systems are designed to evaluate ligand–receptor interactions under controlled conditions and do not reproduce physiological complexity. Therefore, these results do not represent the full complexity of human sensory perception or neurophysiological processes, but rather provide an initial indication of how carrier addition may influence receptor activation patterns.

The reformulated IC 2.0 product showed a distinct receptor activation profile compared to carrier-free IC 0.0 (SD-IC), with reduced bitter taste receptor activation alongside observed muscarinic and dopaminergic GPCR responses under the experimental conditions. However, any (taste) bioactivity or modulation should be confirmed in a sensory panel. These formulation-dependent effects suggest differences in receptor-level responses that may influence sensory-related properties and be relevant for future sensory evaluation studies, although these findings cannot be directly extrapolated to human perception or consumer acceptance.

**Figure 11:**
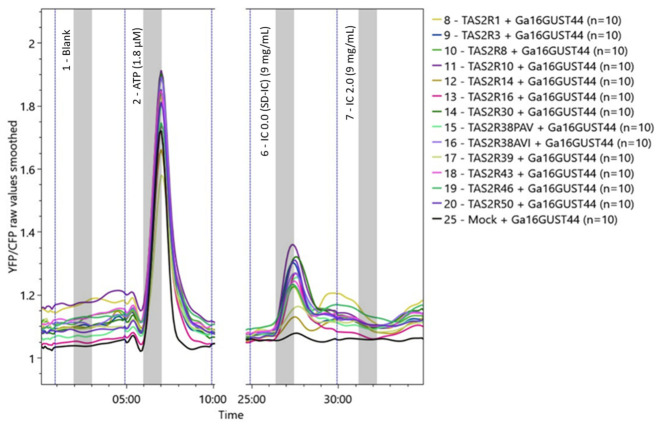
Bioluminescence assay with Venus/Nanoluc BRET ratio curves in response to the injection of 1-Blank, 2-ATP (1.8 μM), 6-IC 0.0 (SD-IC) (9 mg/mL) and 7-IC 2.0 (9 mg/mL). Other injections of unrelated samples are not shown. The curves represent the Mock control (black line) and a selection of 13 commonly active hTAS2R bitter taste receptors. The ATP injection results in a host cell response. The gray areas indicate the 1 min exposure time of the cells to the sample. Time is given in minutes. Ga16GUST44 is the G-alpha protein mediating the calcium response. *n* = 10 corresponds to independent spots (technical replicates) per receptor on the chip.

According to the results previously obtained by our research group, IC 0.0 (SD-IC) had a lower environmental impact than IC 0.0 (FD-IC), and was therefore selected for analysis on the tongue-on-a-chip platform [2,19]. Figure 11 shows the response of 13 selected bitter receptors (TAS2R) and the mock, for IC 0.0 (SD-IC) and IC 2.0 at 9 mg/mL, respectively. Sample concentrations were defined in line with typical preparation scenarios of instant cascara beverages, as previously reported [2]. Each receptor was represented with 10 independent technical replicates spots on each chip.

Notably, significant activation of all 13 printed TAS2Rs was observed for IC 0.0 (SD-IC), whereas no activation was detected for IC 2.0. The activation of all TAS2Rs is very uncommon and should be investigated more deeply to understand whether this represents ligand diversity in the sample or rather some novel generic allosteric activity on this class of bitter receptors. Importantly, the addition of inulin eliminated this response, suggesting a potential modulatory effect on receptor–ligand interactions. This observation is consistent with a possible masking of bitter receptor activation by oligofructose-enriched inulin.

The identification of human TAS2Rs has facilitated the development of specific bitter receptor antagonists, which could be widely applied in foods with undesirable bitter tastes [54]. Several TAS2R antagonists, also known as bitter blockers, have been shown to act at the receptor level, reducing bitter intensity in a limited range of food products [19,54,55,56]. Further studies are needed to identify the specific compounds in cascara responsible for TAS2R activation.

The reduced activation of several bitter receptors in IC 2.0 compared with IC 0.0 (SD-IC) suggests that the presence of oligofructose-enriched inulin may influence the interaction of cascara compounds with receptor systems, with potential implications for perceived bitterness. Inulin may contribute to a bitterness-masking effect in Instant Cascara beverages, potentially through matrix-related interactions, although this requires confirmation through sensory evaluation [57].

It should be noted that inulin-type fructans are not considered direct ligands of taste receptors. Their effects on sensory-related responses are primarily attributed to indirect mechanisms, such as modulation of viscosity and tastant release, as well as interactions with phenolic compounds within the matrix [21,58]. Although oligofructose fractions may contain trace amounts of simple sugars capable of interacting with sweet taste receptors, their contribution is expected to be minor under the experimental conditions tested. In contrast, phenolic compounds present in cascara are well-established ligands of bitter taste receptors (TAS2Rs) and are therefore more likely to drive the receptor-level responses observed [54,55,56].

This hypothesis should be confirmed in future research, taking into account that the sweetness profile of inulin varies depending on its degree of polymerization (DP). Short-chain fractions, also known as fructooligosaccharides (e.g., Synergy 1), exhibit a higher sweetness profile, and their interaction with taste receptors may differ from that of long-chain inulin.

**Figure 12:**
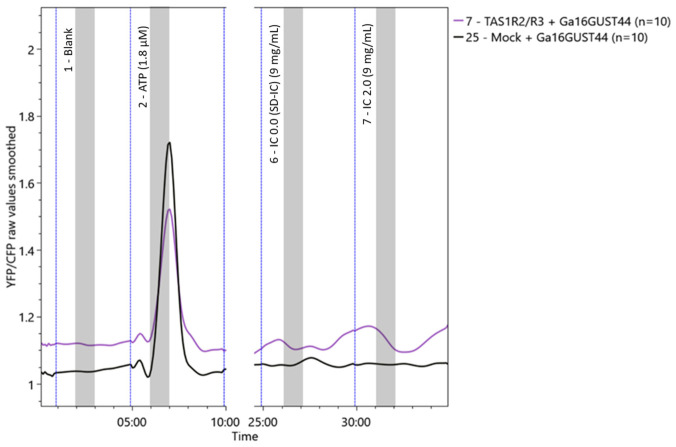
Bioluminescence assay with Venus/Nanoluc BRET ratio curves in response to the injection of 1-Blank, 2-ATP (1.8 μM), 6-IC 0.0 (SD-IC) (9 mg/mL) and 7-IC 2.0 (9 mg/mL). Other injections of unrelated samples are not shown. The curves represent the Mock control (black line) and the sweet heterodimeric receptor hTAS1R2/hTAS1R3. The ATP injection results in a host cell response. The gray areas indicate the 1 min exposure time of the cells to the sample. Time is given in minutes. Ga16GUST44 is the G-alpha protein mediating the calcium response. *n* = 10 corresponds to independent spots (technical replicates) per receptor on the chip.

The response of the heterodimer sweet receptor hTAS1R2/hTAS1R3 was also studied. The activation of TAS1R2/3 on the tongue is a key factor in the perception of sweetness, as these receptors mediate the sweet taste of most natural sugars as well as non-caloric sweeteners [19]. As shown in Figure 12, no activation was detected for either IC 0.0 (SD-IC) or IC 2.0 at 9 mg/mL. The lack of activation suggests that the components of the beverage do not interact with the TAS1R2/3 receptors under the conditions and concentrations tested. Whether this corresponds to perceived sweetness should be assessed through sensory evaluation.

**Figure 13:**
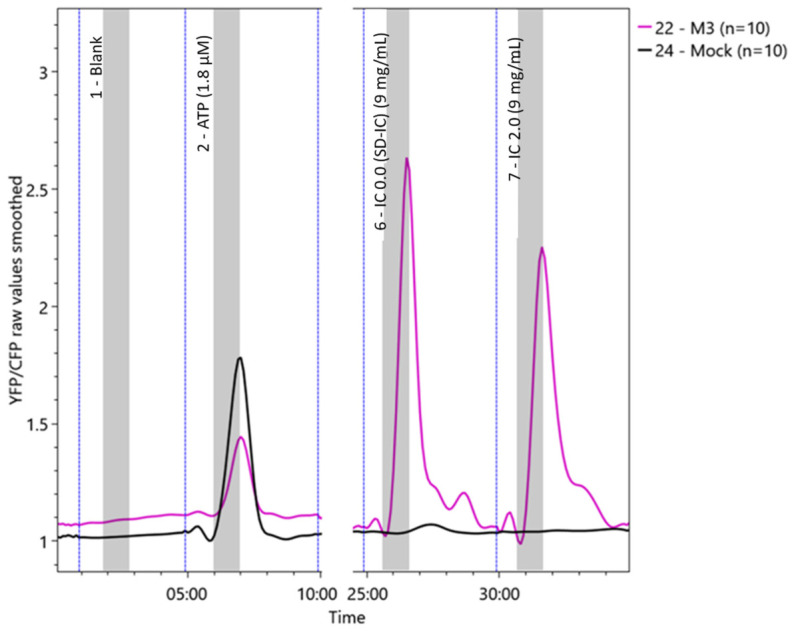
Bioluminescence assay with Venus/Nanoluc BRET ratio curves in response to the sequential injection of 1-Blank, 2-ATP (1.8 μM), 6-IC 0.0 (SD-IC) (9 mg/mL) and 7-IC 2.0 (9 mg/mL). Other injections of unrelated samples are not shown. The curves represent the Mock control (black line) and the muscarinic CHRM3 receptor (M3). The ATP injection results in a host cell response. The gray areas indicate the 1 min exposure time of the cells to the sample. Time is given in minutes. *n* = 10 corresponds to independent spots (technical replicates) per receptor on the chip.

On the same receptor cell array, the response of IC 0.0 (SD-IC) and IC 2.0 to the CHRM3 muscarinic receptor (M3) was also evaluated (Figure 13). Muscarinic acetylcholine receptors (mAChRs) are a family of G-protein-coupled receptors involved in multiple biological processes [59,60]. Among the five subtypes (CHRM1-M5), the M3 muscarinic acetylcholine receptor (CHRM3) has been associated with the regulation of exocrine gland secretions, such as saliva production [60].

As can be seen in Figure 13, both beverages showed a similar signal at the CHRM3 receptor, indicating receptor-level responses in neurotransmitter-associated targets. To our knowledge, this observation has not been previously reported for coffee cascara beverages.

Rubach et al. studied the effects of coffee beverage components on CHRM3 receptor protein expression, finding that chlorogenic acid significantly increased M3 receptor expression by approximately 56% and that caffeine doubled this expression compared to control cells, although receptor activation was not assessed in that study [61].

Response patterns in M3 receptors have also been described for bioactive compounds from other plant-based extracts, such as rooibos [60]. Further studies are needed to identify which specific compounds present in cascara beverages, particularly Instant Cascara (reference beverage and 2.0), are responsible for these receptor-level responses.

**Figure 14:**
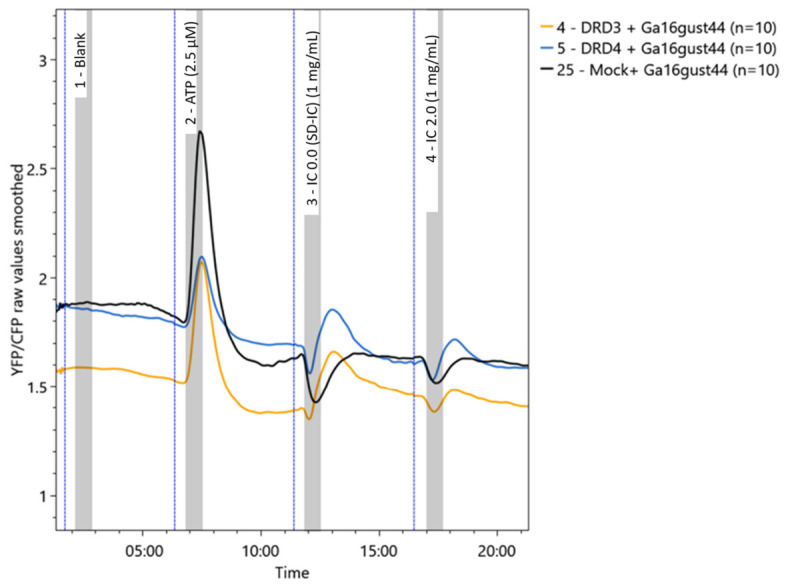
Fluorescence assay with YFP/CFP FRET ratio curves in response to the sequential injection of 1-Blank, 2-ATP (2.5 μM), 3-IC 0.0 (SD-IC) (1 mg/mL) and 4-IC 2.0 (1 mg/mL). The curves represent the Mock control (black line) and the DRD3 and DRD4 dopamine receptors. The ATP injection results in a host cell response. The gray areas indicate the 1 min exposure time of the cells to the sample. Time is given in minutes. The dips may have been caused by sample fluorescence. Ga16GUST44 is the G-alpha protein mediating the calcium response. *n* = 10 corresponds to independent spots (technical replicates) per receptor on the chip.

The effect of beverages on dopamine receptors was evaluated using a chip containing a panel of neurotransmitter receptor GPCRs and a fluorescence-based platform [19]. The results are shown in Figure 14. Dopaminergic receptors are a class of G protein-coupled receptors, but they also exist as a category of ion channel receptors. These receptors are involved in multiple biological processes. Five different GPCR subtypes (D1, D2, D3, D4 and D5) have been described, each with distinct functions and expression patterns in different tissues. D1 and D5 receptors have been associated with processes such as reward, memory, motor activity, and learning, while D2 to D4 receptors have been linked to signaling processes relevant to dopaminergic neuron function and development [62].

As shown in Figure 14, dopamine D3 and D4 receptors responded to both IC 0.0 (SD-IC) and IC 2.0. No signal was detected for the other dopamine receptors under the conditions studied. The D3 receptor is involved in cognition, impulse control, attention and sleep, while the D4 receptor influences cognition, memory, fear, impulse control, attention and sleep [62]. Studies compiled in a 2019 review by Iriondo-DeHond et al. indicate that caffeine has been associated with increased extracellular levels of dopamine [63]. In addition, it has been reported to influence the expression of dopaminergic receptors and transporters, suggesting a potential role in the modulation of dopaminergic signaling pathways.

The muscarinic and dopaminergic receptor responses should be strictly interpreted as receptor-level interactions observed in engineered in vitro systems. Further studies are needed to identify the specific components responsible for these observed receptor-level interactions.

Overall, these results demonstrate that the study objectives were achieved, showing that the incorporation of inulin-type carriers modulates production yield and formulation-dependent in vitro biological and receptor-level responses.

Receptor-based approaches provided complementary mechanistic insights into matrix–receptor interactions, revealing formulation-dependent differences not only in bitterness-related responses but also in other receptor systems, including neurotransmitter-associated targets. These findings support the relevance of this approach to guide future studies addressing sensory-related and formulation-dependent receptor interactions.

Furthermore, the consistency of the antioxidant and anti-inflammatory responses with previous studies, including simulated digestion, supports the robustness of the cascara matrix across experimental conditions.

Together, these results provide a mechanistic basis for future in vivo and human studies, while acknowledging the limitations inherent to in vitro models.

To provide context for the concentrations used in this study, it is important to consider their relation to realistic consumption scenarios. A typical serving of Instant Cascara beverage may involve several grams of powdered product per portion, consistent with preparation conditions previously described in the literature [2], resulting in the intake of moderate amounts of caffeine, methylxanthines, phenolic compounds, and inulin-type carriers as part of the formulation.

However, the concentrations reached at the systemic level are expected to be significantly lower due to digestion, metabolism, and limited bioavailability. Therefore, the concentrations used in this study are primarily intended to explore mechanistic responses rather than to directly reflect physiological exposure conditions.

### 3.4. Limitations of the Study

The findings of this study should be interpreted within the limitations of in vitro models, which do not fully replicate physiological conditions. In particular, the absence of digestion and bioaccessibility assessment limits the direct extrapolation of the results to in vivo situations and prevents the estimation of gastrointestinal stability and systemic exposure of the bioactive compounds. Therefore, interpretations related to the preservation or modulation of these compounds after formulation should be understood within the context of in vitro conditions.

In addition, the absence of carrier-only control groups limits the ability to fully disentangle the specific contribution of the cascara matrix, the inulin-type carriers, and their potential interactions in the observed biological and receptor-level responses.

Therefore, while formulation-dependent differences are observed, the relative contribution of matrix effects and compositional variations cannot be completely separated under the experimental conditions used.

Furthermore, although the oligofructose-enriched carrier contains a fraction of free mono- and disaccharides, no activation of sweet taste receptors was detected, suggesting a limited impact of this fraction in the receptor-based assays.

Moreover, receptor-based assays reflect interaction patterns under controlled conditions and do not directly represent human sensory perception or physiological responses. These aspects should be addressed in future studies, including digestion models, carrier-specific controls, sensory evaluation, and in vivo validation.

## 4. Conclusions

Instant Cascara formulations, with and without inulin-type carriers, exhibited relevant in vitro biological activities, including antioxidant, anti-inflammatory, antiproliferative, chemoprotective, and pro-apoptotic effects, without evidence of genotoxicity under the conditions tested.

The incorporation of inulin-type carriers influenced production yield and modulated formulation-dependent responses.

Overall, these results support the use of inulin-type carriers as formulation tools and indicate that IC 2.0 provides a favorable balance between production yield, in vitro biological activity, and a distinct receptor-level response profile.

This balance may be associated with a reduced contribution of bitterness-related compounds, such as caffeine, together with the preservation of other bioactive components contributing to the observed biological responses.

In addition, the observed receptor-level differences are consistent with formulation-dependent variations in the effective concentration of bioactive compounds, with caffeine likely acting as a dominant contributor due to its higher relative abundance.

## Figures and Tables

**Table 1:** Definition of Instant Cascara formulations.

Abbreviation	Description
IC 0.0 (FD-IC)	Freeze-dried Instant Cascara without carrier
IC 0.0 (SD-IC)	Spray-dried Instant Cascara without carrier
IC 1.0	Spray-dried Instant Cascara containing long-chain inulin (Orafti® HPX)
IC 2.0	Spray-dried Instant Cascara containing oligofructose-enriched inulin (Orafti® Synergy1)

**Table 2:** Phytochemical composition (methylxanthines and phenolic compounds) of IC 1.0 and IC 2.0.

Analyzed Compounds	IC 1.0	IC 2.0	*p* < 0.05
**Methylxanthines**			
Caffeine (mg/g of dried sample)	7.41 ± 0.04	5.58 ± 0.10	*
Theobromine (µg/g of dried sample)	21.38 ± 1.07	21.33 ± 1.38	
Theophylline (µg/g of dried sample)	8.17 ± 0.89	9.95 ± 0.77	
Total (mg/g of dried sample)	7.44	5.62	
**Phenolic compounds**			
Isoflavones			
Epicatechin (µg/g of dried sample)	9.93 ± 0.65	10.46 ± 0.65	
Catechin hexoside (µg/g of dried sample)	96.86 ± 4.34	94.87 ± 3.85	
Catechin dimer	14.14 ± 0.68	12.57 ± 0.42	*
Mangiferin (µg/g of dried sample)	634.96 ± 22.20	611.38 ± 12.27	
Rutin (µg/g of dried sample)	96.33 ± 2.72	101.39 ± 3.39	
Total (mg/g of dried sample)	0.85	0.83	
Hydroxycinnamic acids			
*p*-coumaroylquinic acid (µg/g of dried sample)	223.65 ± 10.02	218.98 ± 8.88	
Caffeoylquinic acid (µg/g of dried sample)	688.53 ± 27.92	647.09 ± 17.02	
3-O-caffeoylquinic acid (chlorogenic acid) (µg/g of dried sample)	4933.26 ± 279.35	4756.53 ± 145.49	
4-O-feruloylquinic acid (µg/g of dried sample)	10.93 ± 0.33	9.84 ± 0.64	
5-O-feruloylquinic acid (µg/g of dried sample)	669.13 ± 26.55	623.10 ± 6.01	*
3,4-di-O-caffeoylquinic acid (µg/g of dried sample)	259.36 ± 5.74	261.80 ± 7.01	
4,5-di-O-caffeoylquinic acid (µg/g of dried sample)	114.06 ± 9.95	116.56 ± 5.54	
Total (mg/g of dried sample)	6.9	6.63	
Anthocyanins			
Cyanidin-3-O-glucoside (µg/g of dried sample)	N.D.	N.D.	
Cyanidin-3-O-rutinoside (µg/g of dried sample)	N.D.	N.D.	
Total (mg/g of dried sample)	N.D.	N.D.	
TOTAL (mg/g of dried sample)	15.19	13.08	

Total values correspond to the sum of the mean concentrations of individual compounds ± SD. Results are expressed in μg/g dry weight and normalized to the total powder mass. Each sample was analyzed in triplicate. N.D.: not detected (the compound was below the detection limit under the experimental conditions used). Asterisks indicate a significant difference for both samples studied (*t*-Student test, * *p* < 0.05).

## Data Availability

The original contributions presented in this study are included in the article/Supplementary Materials. Further inquiries can be directed to the corresponding author.

## Supplementary Materials

The following supporting information can be downloaded at https://www.mdpi.com/article/10.3390/nu18121932/s1, Document S1: Technical datasheet for Orafti^®^ HPX; Document S2: Technical datasheet for Orafti^®^ Synergy1; Figure S1: Effect of IC 1.0 and IC 2.0 (mg/mL) on CCD-18 cell viability (24 h); Figure S2: Effect of IC 1.0 and IC 2.0 (mg/mL) on Caco-2 cell viability (24 h); Figure S3: Effect of IC 1.0 and IC 2.0 (mg/mL) on RAW 264.7 cell viability (24 h); Table S1: Receptor activation responses (fluorescence assay) of selected receptors in the tongue/gut-on-a-chip platform to Instant Cascara formulations (IC 0.0 (SD-IC) and IC 2.0); Table S2: Receptor activation responses (bioluminescence assay) of taste-related and chemesthetic receptors in the tongue/gut-on-a-chip platform following exposure to Instant Cascara formulations (IC 0.0 (SD-IC) and IC 2.0); Table S3: Statistical comparison among IC 0.0 (FD-IC), IC 0.0 (SD-IC), IC 1.0 and IC 2.0 formulations in selected in vitro assays.

## Abbreviations

The following abbreviations are used in this manuscript:

BaP	Benzo(a)pyrene
BRET	Bioluminescence resonance energy transfer
CYP450	Cytochrome P450
DCFH-DA	2,7-dichlorofluorescein diacetate
DMEM	Dulbecco’s modified Eagle Medium
DMSO	Dimethyl sulfoxide
EFSA	European Food Safety Authority
ESI	Electrospray ionization
FBS	Fetal bovine serum
FD-IC	Freeze-drying Instant Cascara
FRET	Förster resonance energy transfer
GPCR	G-protein-coupled receptors
IARC	International Agency for Research on Cancer
IC	Instant Cascara
IC 0.0	Instant Cascara without carrier
IC 1.0	Instant Cascara with long-chain inulin
IC 2.0	Instant Cascara with oligofructose-enriched inulin
iNOS	Nitric oxide synthase
LMP	Low-melting-point agarose
LPS	Lipopolysaccharide
MAChRs	Muscarinic acetylcholine receptors
MTT	3-(4,5-dimethylthiazol-2-yl)-2,5-diphenyltetrazolium bromide
NO	Nitric oxide
PBS	Phosphate-buffered saline
PhIP	2-amino-1-methyl-6-phenylimidazo[4,5-b]-pyridine
PI	Propidium iodide
ROS	Reactive oxygen species
SD	Standard deviation
SD-IC	Spray-drying Instant Cascara
tBOOH	Tert-butyl hydroperoxide
