# Supplementary material for: Instant Cascara Beverages with Inulin-Type Carriers: Production Yield, In Vitro Biological Activity and Receptor-Level Responses
Source: Nutrients. 2026 Jun 15;18(12):1932. doi: 10.3390/nu18121932 (PMC13306141; doi:10.3390/nu18121932)
Supplement: Supplementary file 1 [file nutrients-18-01932-s001.zip › nutrients-4316050-supplementary.pdf]

nutrients

MDPI

Article

Instant Cascara Beverages with Inulin-Type Carriers: Production Yield, In Vitro Biological Activity and Receptor-Level Responses

Vanesa Sánchez-Martín [1], Marta B. López-Parra [1], Margriet Roelse [2], Amaia Iriondo-DeHond [3], Paloma Morales [3], Ana I. Haza [3], Maarten A. Jongsma [2] and María Dolores del Castillo [1,*]

[1] Instituto de Investigación en Ciencias de la Alimentación (CIAL), Consejo Superior de Investigaciones Científicas (CSIC), Universidad Autónoma de Madrid (UAM), 28049 Madrid, Spain; vanesa.s@csic.es (V.S.-M.); martab.lopez.parra@gmail.com (M.B.L.-P.)

[2] Business Unit Bioscience, Wageningen Plant Research, Droevendaalsesteeg 1, 6708 PB Wageningen, The Netherlands; margriet@insectsense.com (M.R.); maarten.jongsma@wur.nl (M.A.J.)

[3] Sección Departamental de Nutrición y Ciencia de los Alimentos, Departamento de Nutrición y Ciencia de los Alimentos, Facultad de Veterinaria, Universidad Complutense, 28040 Madrid, Spain; amaiairi@ucm.es (A.I.-D.); pmorales@ucm.es (P.M.); hanais@ucm.es (A.I.H.)

* Correspondence: mdolores.delcastillo@csic.es; Tel.: +34-910017900

Supplementary Document S1. Technical datasheet for Orafti® HPX.

beneo
connecting nutrition and health

Orafti®HPX

Description

- Orafti®HPX is a food ingredient (powder) consisting of long-chain chicory inulin. Compared to the standard Orafti®HP, Orafti®HPX has higher gel strength in fat substitution applications at higher temperatures.
- Long-chain inulin consists of polysaccharides composed of fructose units linked together by ß-(2,1)-linkages. Almost every fructose chain is terminated by a glucose unit. Long-chain inulin is produced by removal of the shorter chain inulin from chicory inulin. The number of fructose and glucose units (Degree of polymerization = DP) ranges mainly from 5 to 60.

Specifications

Physical and Chemical Parameters

Parameter	Limit	Unit	Reference method[1]	Frequency
**Inulin**	min 99.5	g/100 g d.m.	AOAC 997.08	Each batch[2]
**Glucose + fructose + sucrose**	max 0.5	g/100 g d.m.	AOAC 997.08	Each batch[2]
**Dry matter** (d.m.)	97 ± 2	g/100 g	Vacuum (<35 mbar, 70 °C, 20 h)	Each batch
**pH** (10 g/100 g)	6 ± 1		Potentiometric (20 °C)	Each batch
**Conductivity** (15 g/100 g)	max 250	µS/cm	ICUMSA GS2/3/9-17, adapted	Each batch
**Ash** (sulphated)	max 0.2	g/100 g d.m.	ICUMSA GS3/4/7/8-11, adapted	Monitoring
**Arsenic** (total)	max 0.03	mg/kg	ICP-MS	Monitoring
**Lead**	max 0.02	mg/kg	ICP-MS	Monitoring
**Mercury**	max 0.01	mg/kg	AAS	Monitoring
**Cadmium**	max 0.01	mg/kg	ICP-MS	Monitoring

1 or validated equivalent
2 CoA: % carbohydrates (HPLC)

BENEO-Orafti S.A. · Office Tienen: Aandorenstraat 1 · 3300 Tienen · Belgium · Phone +32 16 801 301 · Fax +32 16 801 308
Corporate Seat: Rue Louis Maréchal 1 · 4360 Oreye · Belgium · www.beneo.com · contact@beneo.com · RPM Liège 0413.631.556

SÜDZUCKER GROUP

beneo
connecting nutrition and health

Microbiological Parameters

Parameter	Limit	Unit	Method[1]	Frequency
**Total mesophilic bacteria (aerobes)**	max 1 000	cfu/g	ICUMSA GS2/3-41	Each batch
**Yeasts**	max 20	cfu/g	ICUMSA GS2-47	Each batch
**Moulds**	max 20	cfu/g	ICUMSA GS2-47	Each batch
**Thermophilic aerobic spores**	max 1 000	cfu/g	ICUMSA GS2/3-49	Each batch
**Enterobacteriaceae** (incl. coliforms, E. coli)	< 1	cfu/g	ISO 21528-2	Each batch
**Clostridia** (incl. C. perfringens)	< 1	cfu/g	ISO 15213	Monitoring
**Bacillus cereus**	max 10	cfu/0.1 g	ISO 7932	Monitoring
**Coagulase-positive staphylococci**	Not detected	/g	ISO 6888	Monitoring
**Salmonella**	Not detected	/375 g	ISO 6579	Monitoring
**Listeria monocytogenes**	Not detected	/25 g	ISO 11290	Monitoring

[1] or acknowledged and validated equivalent

Additional Information

**Carbohydrates**	Min. 99.5 g/100 g d.m.<br>(calculated as dry matter minus ash minus protein minus fat)
**DP**	Average DP ≥ 23; 99 % of the inulin has a DP ≥ 5
**Appearance***	Fine, white to slightly yellow powder
**Behaviour***	Hygroscopic
**Taste***	Neutral
**Solubility in water***	< 1 g/L at 25 °C
**Bulk density***	530 g/L ± 50 g/L
**Proposed labelling (EU)**	Inulin
(other countries)	Information available upon request
**Packaging**	Paper bags, Big Bags; both on pallets
**Recommended storage conditions**	Temperature < 25 °C,<br>Relative humidity below 60%
**Minimum durability**	5 years in bags from date of production under recommended storage conditions in its original unopened packaging<br>3 years in Big Bags from date of production under recommended storage conditions in its original unopened packaging
**Safety precautions**	Orafti®HPX is a fine powder and can cause dust explosions when mixed with air (typical for powder).
**Certification and Compliance**	Kosher (certificate available upon request)<br>Halal (certificate available upon request)<br>Suitable for vegetarians & vegans<br>Suitable for gluten-free products: gluten ≤ 10 mg/kg<br>Orafti®HPX is not produced from ingredients or using processing aids that would require allergen labelling as laid down in Regulation (EU) No 1169/2011.<br><br>Orafti®HPX is produced in Belgium or Chile in compliance with applicable Belgian, Chilean and European Food Law (e.g. Regulation (EC) No 178/2002, Regulation (EC) No 852/2004) and a GFSI-recognized food safety standard.

Information relevant for Nutrition Declaration

Nutritional information provided in the table shall enable food manufacturers to calculate the contribution of Orafti®HPX in their food products in compliance with the applicable EU/US regulations. More detailed information is available upon request.

Any nutritional claim made using Orafti®HPX is based on an appropriate use level (grams/serving) of commercial product. Please consult your local BENEO contact with any questions.

Nutrient	Unit per 100 g	Typical Value	
		EU	US
**Energy/calories**[1]	kJ/kcal	776/194	Not applicable/194
**Total Fat**[2]	g	**Negligible**[3]	
Saturates	g	Negligible[3]	
**Total carbohydrate**[4]	g	Not applicable	97
**Carbohydrate**[5]	g	0	Not applicable
(Total) sugars	g	0	0
Added sugars	g	Not applicable	Not applicable[6]
**Dietary fibre**[7] (AOAC 997.08)	g	97	97
**Protein**	g	**Negligible**[3]	
**Salt** (sodium)	g	**Negligible**[3]	
**Vitamins, minerals**	g	**Negligible**[3]	

1 Applying the energy conversion factor of 8 kJ/g or 2 kcal/g as laid down for all fibres in the EU and for all soluble non-digestible carbohydrates in the US
2 Applicable to US: trans fats and cholesterol also negligible
3 Negligible means "0" according to applicable rounding rules
4 Applicable to US: Total carbohydrate includes dietary fibre
5 Applicable to EU: Carbohydrate does not include dietary fibre
6 Further information available upon request
7 EU/US dietary fibre definition: non-digestible carbohydrate oligo- or polymers with three or more monomeric units (DP ≥ 3); excludes inulobiose

Document Code	Version	Valid from
HPX A4-07	007	01-01-2023

Disclaimer To the best of our knowledge, the information in this sheet is reliable. BENEO-Orafti S.A. warrants all parameters; those marked with an asterisk (*) cannot be subject of complaints.

Supplementary Document S2. Technical datasheet for Orafti® Synergy1.

Product Sheet

beneo
connecting nutrition and health

Orafti®Synergy1

Description

- Orafti®Synergy1 is a food ingredient (powder) consisting of a unique combination of chicory inulin fractions with selected chain lengths. In Orafti®Synergy1 shorter chain inulin (oligofructose) is combined with longer chain inulin in essentially equal amounts.
- Inulin consists of oligo- and polysaccharides composed of fructose units linked together by ß-(2,1)-linkages. Almost every fructose chain is terminated by a glucose unit. The number of fructose and glucose units in inulin (Degree of Polymerization = DP) ranges mainly between 2 and 60.
- Shorter chain inulin (oligofructose) consists of oligosaccharides obtained by partial enzymatic hydrolysis from inulin. Part of shorter chain inulin is terminated by a glucose unit. The degree of polymerization is less than 10.
- Longer chain inulin consists of inulin of which shorter fractions were removed. Almost every molecule is terminated by a glucose unit and has a DP of 10 or higher.

Specifications

Physical and Chemical Parameters

Parameter	Limit	Unit	Reference method[1]	Frequency
**Inulin**	92 ± 2	g/100 g d.m.	AOAC 997.08	Each batch[2]
**Glucose + fructose + sucrose**	8 ± 2	g/100 g d.m.	AOAC 997.08	Each batch[2]
**Dry matter** (d.m.)	97 ± 2	g/100 g	Vacuum (<35 mbar, 70 °C, 20 h)	Each batch
**pH** (10 g/100 g)	6 ± 1		Potentiometric (20 °C)	Each batch
**Conductivity** (15 g/100 g)	max 250	µS/cm	ICUMSA GS2/3/9-17, adapted	Each batch
**Ash** (sulphated)	max 0.2	g/100 g d.m.	ICUMSA GS3/4/7/8-11, adapted	Monitoring
**Arsenic** (total)	max 0.03	mg/kg	ICP-MS	Monitoring
**Lead**	max 0.02	mg/kg	ICP-MS	Monitoring
**Mercury**	max 0.01	mg/kg	AAS	Monitoring
**Cadmium**	max 0.01	mg/kg	ICP-MS	Monitoring

1 or validated equivalent
2 CoA: % carbohydrates (HPLC)

Microbiological Parameters

Parameter	Limit	Unit	Method[1]	Frequency
Total mesophilic bacteria (aerobes)	max 1 000	cfu/g	ICUMSA GS2/3-41	Each batch
Yeasts	max 20	cfu/g	ICUMSA GS2-47	Each batch
Moulds	max 20	cfu/g	ICUMSA GS2-47	Each batch
Thermophilic aerobic spores	max 1 000	cfu/g	ICUMSA GS2/3-49	Each batch
Enterobacteriaceae (incl. coliforms, E. coli)	< 1	cfu/g	ISO 21528-2	Each batch
Clostridia (incl. C. perfringens)	< 1	cfu/g	ISO 15213	Monitoring
Bacillus cereus	max 10	cfu/0.1 g	ISO 7932	Monitoring
Coagulase-positive staphylococci	Not detected	/g	ISO 6888	Monitoring
Salmonella	Not detected	/375 g	ISO 6579	Monitoring
Listeria monocytogenes	Not detected	/25 g	ISO 11290	Monitoring

[1] or acknowledged and validated equivalent

Additional Information

**Carbohydrates**	Min. 99.5 g/100 g d.m.<br>(calculated as dry matter minus ash minus protein minus fat)
**DP**	shorter chain inulin: DP 3-9, (50±10)% (HPAEC)<br>longer chain inulin: DP≥10, (50±10)% (HPAEC)
**Appearance***	Fine, white to slightly yellow powder
**Behaviour***	Hygroscopic
**Taste***	Slightly sweet
**Solubility in water***	approx. 50 g/L at 25°C
**Bulk density***	620 g/L ± 50 g/L
**Proposed labelling (EU)**	"Oligofructose enriched inulin" or "inulin, oligofructose"
(other countries)	Information available upon request
**Packaging**	Paper bags, Big Bags; both on pallets
**Recommended storage conditions**	Temperature < 25 °C,<br>Relative humidity below 60%
**Minimum durability**	5 years in bags from date of production under recommended storage conditions in its original unopened packaging<br>3 years in Big Bags from date of production under recommended storage conditions in its original unopened packaging
**Safety precautions**	Orafti®Synergy1 is a fine powder and can cause dust explosions when mixed with air (typical for powder).
**Certification and Compliance**	Kosher (certificate available upon request)<br>Halal (certificate available upon request)<br>Suitable for vegetarians & vegans<br>Suitable for gluten-free products: gluten ≤ 10 mg/kg<br>Orafti®Synergy1 is not produced from ingredients or using processing aids that would require allergen labelling as laid down in Regulation (EU) No 1169/2011.<br><br>Orafti®Synergy1 is produced in Belgium or Chile in compliance with applicable Belgian, Chilean and European Food Law (e.g. Regulation (EC) No 178/2002, Regulation (EC) No 852/2004) and a GFSI-recognized food safety standard.

Information relevant for Nutrition Declaration

Nutritional information provided in the table shall enable food manufacturers to calculate the contribution of Orafti®Synergy1 in their food products in compliance with the applicable EU/US regulations. More detailed information is available upon request.

Any nutritional claim made using Orafti®Synergy1 is based on an appropriate use level (grams/serving) of commercial product. Please consult your local BENEO contact with any questions.

Nutrient	Unit per 100 g	Typical Value	
		EU	US
**Energy/calories**[1]	kJ/kcal	866/214	Not applicable/214
**Total Fat**[2]	g	Negligible[3]	
Saturates	g	Negligible[3]	
**Total carbohydrate**[4]	g	Not applicable	97
**Carbohydrate**[5]	g	10	Not applicable
(Total) sugars	g	10	10
Added sugars	g	Not applicable	Not applicable[6]
**Dietary fibre**[7] (AOAC 997.08)	g	87	87
**Protein**	g	Negligible[3]	
**Salt** (sodium)	g	Negligible[3]	
**Vitamins, minerals**	g	Negligible[3]	

1 Applying the energy conversion factor of 8 kJ/g or 2 kcal/g as laid down for all fibres in the EU and for all soluble non-digestible carbohydrates in the US
2 Applicable to US: trans fats and cholesterol also negligible
3 Negligible means "0" according to applicable rounding rules
4 Applicable to US: Total carbohydrate includes dietary fibre
5 Applicable to EU: Carbohydrate does not include dietary fibre
6 Further information available upon request
7 EU/US dietary fibre definition: non-digestible carbohydrate oligo- or polymers with three or more monomeric units (DP ≥ 3); excludes inulobiose

Document Code	Version	Valid from
Synergy1 A4-20	007	01-01-2023

Disclaimer To the best of our knowledge, the information in this sheet is reliable. BENEO-Orafti S.A. warrants all parameters; those marked with an asterisk (*) cannot be subject of complaints.

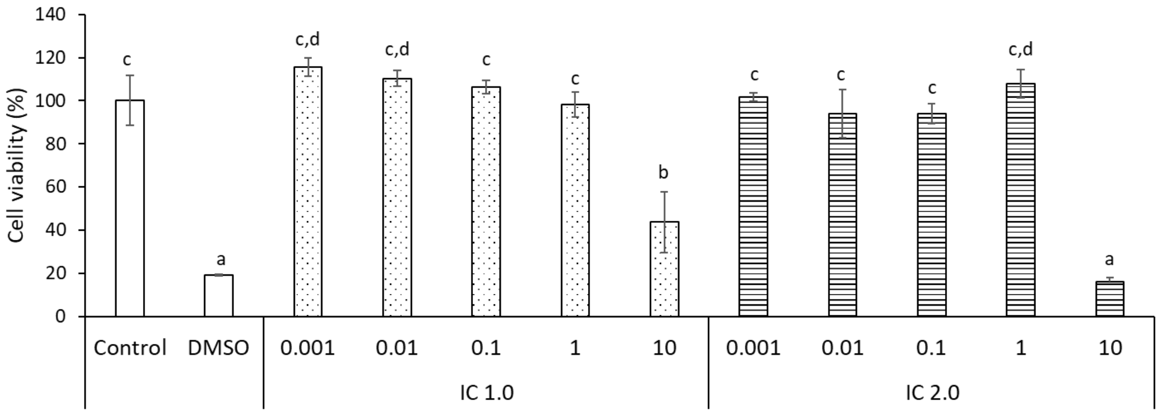

Supplementary Figure S1. Effect of IC1.0 and IC 2.0 (mg/ml) on CCD-18 cell viability (24 h). Control refers to untreated cells and DMSO (50%) was used as a death control. Data are expressed as mean ± SD (n = 3). Different letters indicate significant differences (Tukey's test; $p < 0.05$).

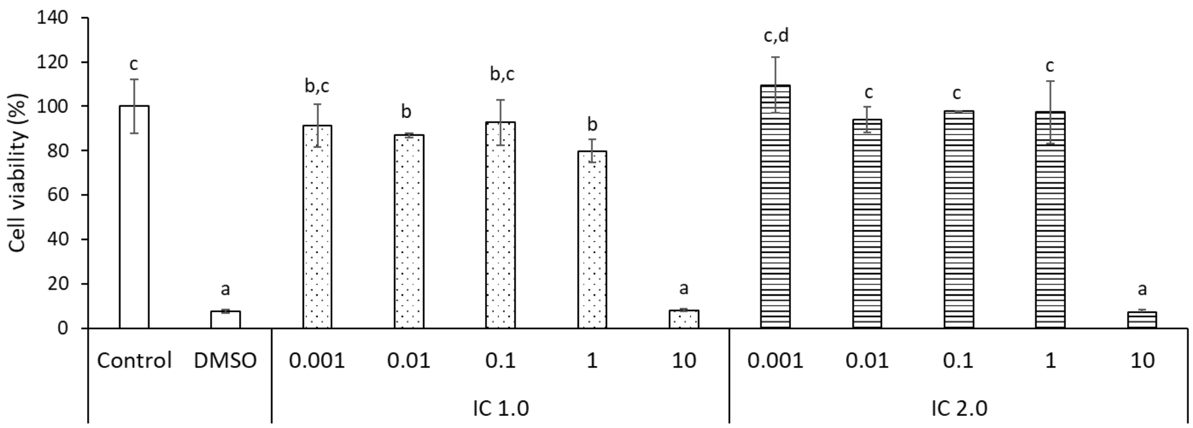

Supplementary Figure S2. Effect of IC 1.0 and IC 2.0 (mg/ml) on Caco-2 cell viability (24 h). Control refers to untreated cells and DMSO (50%) was used as a death control. Data are expressed as mean ± SD (n = 3). Different letters indicate significant differences (Tukey's test; $p < 0.05$).

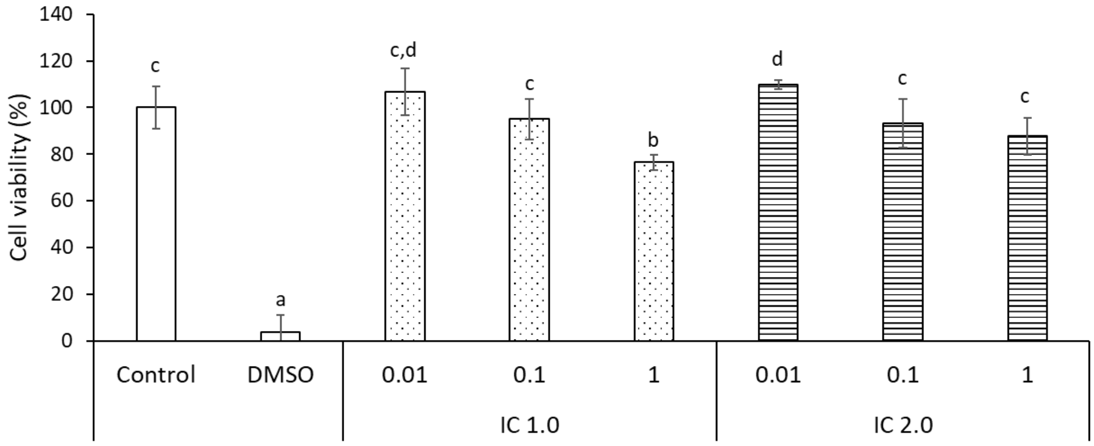

Supplementary Figure S3. Effect of IC 1.0 and IC 2.0 (mg/ml) on RAW 264.7 cell viability (24 h). Control refers to untreated cells and DMSO (50%) was used as a death control. Data are expressed as mean ± SD (n = 3). Different letters indicate significant differences (Tukey's test; $p < 0.05$).

Supplementary Table S1. Receptor activation responses (fluorescence assay) of selected receptors in the tongue/gut-on-a-chip platform to Instant Cascara formulations (IC 0.0 (SD-IC) and IC 2.0).

Receptor	IC 0.0 (SD-IC) 1 mg/ml	IC 2.0 1 mg/ml
CCKBR	N	N
DRD1 + Ga16GUST44	N	N
DRD2 + Ga16GUST44	N	N
DRD3 + Ga16GUST44	Y	Y
DRD4 + Ga16GUST44	Y	Y
DRD5 + Ga16GUST44	N	N
FFAR1	N	N
FFAR2	N	N
GSGR	N	N
HTR1B + Ga16GUST44	N	N
HTR2A	N	N
HTR2B	N	N
HTR2C	Y	Y
M2	N	N
M3	Y	Y
M4	N	N
M5	N	N
NPY1 + Ga16GUST44	N	N
NPY2 + Ga16GUST44	N	N
NPY4 + Ga16GUST44	N	N
NPY5 + Ga16GUST44	N	N
GLP1	N	N
Mock	N	N
Mock + Ga16GUST44	N	N
Sensor control	Y[1]	Y[1]

Activation (receptor-sample interaction) is indicated as Y (yes) or N (no). [1] A positive signal in the sensor control implies the presence of sample fluorescence.

Supplementary Table S2. Receptor activation responses (bioluminescence assay) of taste-related and chemesthetic receptors in the tongue/gut-on-a-chip platform following exposure to Instant Cascara formulations (IC 0.0 (SD-IC) and IC 2.0).

Receptor	IC 0.0 (SD-IC) 9 mg/ml	IC 2.0 9 mg/ml
TRPA1	N	N
TRPC5	N	N
TRPV1	Y	Y
TRPM8	N	N
CasR	N	N
TAS1R2/R3 (Sweet) + Ga16GUST44	N	N
TAS2R1 + Ga16GUST44	Y	N
TAS2R3 + Ga16GUST44	Y	N
TAS2R8 + Ga16GUST44	Y	N
TAS2R10 + Ga16GUST44	Y	N
TAS2R14 + Ga16GUST44	Y	N

TAS2R16 + Ga16GUST44	Y	N
TAS2R30 + Ga16GUST44	Y	N
TAS2R38PAV + Ga16GUST44	Y	N
TAS2R38AVI + Ga16GUST44	Y	N
TAS2R39 + Ga16GUST44	Y	N
TAS2R43 + Ga16GUST44	Y	N
TAS2R46 + Ga16GUST44	Y	N
TAS2R50 + Ga16GUST44	Y	N
GLP1	N	N
M3 (muscarine)	Y	Y
HTR2C	N[1]	N[1]
Mock	N	N
Mock + Ga16GUST44	N	N
Sensor control	N	N

Activation (receptor-sample interaction) is indicated as Y (yes) or N (no). [1] There was an inconclusive peak for HTR2C. The array was exposed to other samples prior to these samples. These prior exposures triggered very high HTR2C responses. These may have caused receptor exhaustion for HTR2C and explain the difference with the previous experiment. A positive signal in the sensor control implies the presence of sample fluorescence.

Supplementary Table S3. Statistical comparison among IC 0.0 (FD-IC), IC 0.0 (SD-IC), IC 1.0 and IC 2.0 formulations in selected *in vitro* assays.

**Assay**	**IC 0.0 (FD-IC)**	**IC 0.0 (SD-IC)**	**IC 1.0**	**IC 2.0**
Genotoxicity	a	a	a	a
Chemoprotection	a	a	a	a
Proliferation (CCD-18 cells, 0.1 mg/ml)	a	a	a	a
Proliferation (Caco-2 cells, 0.1 mg/ml)	a	a	a	a
Cell cycle (0.5 mg/ml)	b	a	a	b
Cell cycle (1 mg/ml)	b	a	a	b
Cell cycle (1.5 mg/ml)	d	b	a	c
Cell cycle (2 mg/ml)	c	b	a	b
Apoptosis (0.5 mg/ml)	c	b	a	c
Apoptosis (1 mg/ml)	b	b	a	c
Apoptosis (1.5 mg/ml)	d	b	a	c
Apoptosis (2 mg/ml)	c	b	a	b

Different letters indicate statistically significant differences among formulations within each row ($p < 0.05$). For genotoxicity and chemoprotection assays, no significant differences were observed between formulations across the tested concentration range.
